# The maintenance of standing genetic variation: Gene flow vs. selective neutrality in Atlantic stickleback fish

**DOI:** 10.1111/mec.16269

**Published:** 2021-11-25

**Authors:** Quiterie Haenel, Laurent Guerard, Andrew D. C. MacColl, Daniel Berner

**Affiliations:** ^1^ Zoology Department of Environmental Sciences University of Basel Basel Switzerland; ^2^ Imaging Core Facility Biozentrum, University of Basel Basel Switzerland; ^3^ School of Life Sciences University of Nottingham Nottingham UK

**Keywords:** allele frequency, ancestor, evolutionary genomics, *Gasterosteus aculeatus*, migration–selection balance, North Uist, purifying selection, whole‐genome sequencing

## Abstract

Adaptation to derived habitats often occurs from standing genetic variation. The maintenance within ancestral populations of genetic variants favourable in derived habitats is commonly ascribed to long‐term antagonism between purifying selection and gene flow resulting from hybridization across habitats. A largely unexplored alternative idea based on quantitative genetic models of polygenic adaptation is that variants favoured in derived habitats are neutral in ancestral populations when their frequency is relatively low. To explore the latter, we first identify genetic variants important to the adaptation of threespine stickleback fish (*Gasterosteus aculeatus*) to a rare derived habitat—nutrient‐depleted acidic lakes—based on whole‐genome sequence data. Sequencing marine stickleback from six locations across the Atlantic Ocean then allows us to infer that the frequency of these derived variants in the ancestral habitat is unrelated to the likely opportunity for gene flow of these variants from acidic‐adapted populations. This result is consistent with the selective neutrality of derived variants within the ancestor. Our study thus supports an underappreciated explanation for the maintenance of standing genetic variation, and calls for a better understanding of the fitness consequences of adaptive variation across habitats and genomic backgrounds.

## 1 INTRODUCTION

In eukaryotes, adaptation of populations to novel ecological conditions often occurs from standing genetic variation (SGV), that is, selectively relevant variation pre‐existing in the ancestor (Barrett & Schluter, 2008; Hermisson & Pennings, 2005; Matuszewski et al., 2015; Messer & Petrov, 2013; Orr & Betancourt, 2001). A puzzle, however, is how SGV is maintained in the ancestor (Yeaman, 2015): if genetic variants are favoured by selection in a novel, derived habitat, should they not be unfavourable and hence eliminated by purifying selection in the ancestral habitat? One solution to this paradox is that genetic variants favoured in the derived habitat are maintained as SGV in the ancestor by continued hybridization (and hence gene flow) between derived and ancestral populations, thus counteracting the selective removal of these variants in the latter (Barrett & Schluter, 2008; Bolnick & Nosil, 2007; Colosimo et al., 2005; Galloway et al., 2020; Schluter & Conte, 2009; Yeaman & Whitlock, 2011). An alternative idea is that variants beneficial within the novel habitat are selectively neutral in the ancestral population when their frequency is relatively low. While this must obviously hold for recessive variants (Barrett & Schluter, 2008), quantitative genetic models suggest that when the traits under selection are highly polygenic (i.e., influenced by a great number of loci), adaptive divergence may generally occur primarily via the establishment of linkage disequilibrium among alleles and involve only relatively subtle (or at least incomplete) allele frequency differentiation (Kremer & Le Corre, 2012; Latta, 1998; Le Corre & Kremer, 2012). In this case, SGV could persist in the ancestor simply because there is no purifying selection to complete its elimination. The relative importance of these two not mutually exclusive explanations for the maintenance of SGV, gene flow–selection balance and selective neutrality, remains unknown and has, to the best of our knowledge, not been subject to empirical investigation. An obstacle for doing so is that organismal systems are required in which adaptive genetic variation can be detected and quantified in both derived and ancestral populations simultaneously.

**FIGURE 1:**
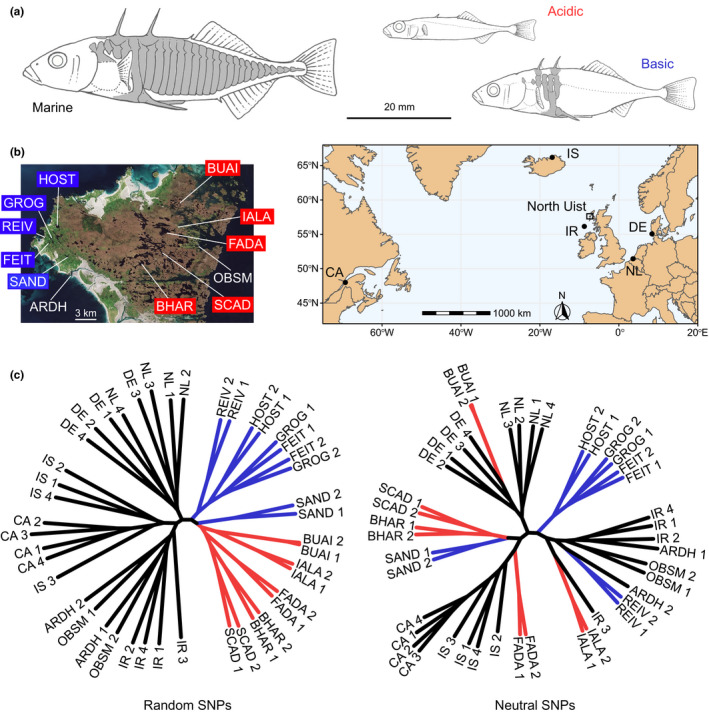
(a) Typical stickleback ecomorphs from marine, acidic freshwater and standard freshwater (here called “basic”) habitats, highlighting the particularly strong reduction in bony armour and body size in acidic stickleback. Key external skeletal elements (dorsal spines, lateral plates, pelvic complex) are shaded in grey. (b) Image of North Uist (left), indicating the acidic (red) and basic (blue) lakes from which freshwater stickleback were sampled. The sites ARDH and OBSM represent locations at which marine stickleback were collected. The other five Atlantic marine sample sites are located in the map (right; North Uist is indicated by the small rectangle). (c) Unrooted maximum‐likelihood phylograms showing the genetic similarity among 44 total marine, acidic and basic stickleback individuals. The left tree is based on 200,000 SNPs selected at random across the genome, whereas the right tree uses 120,448 SNPs filtered to be little influenced by selection (i.e., exhibiting low allele frequency differentiation in both marine–freshwater and acidic–basic genome scans, and located in chromosome regions showing high recombination rates)

We here perform such an investigation in threespine stickleback fish (*Gasterosteus aculeatus*) by focusing on genetic variation promoting the adaptation of populations to acidic freshwater habitats after the recent (postglacial) colonization of these habitats by ancestral marine stickleback. Adaptation to acidic waters probably involves numerous traits, but particularly obvious elements include the reduction of external skeletal armour and body size in some acid‐adapted stickleback populations relative to their ancestor (and to standard freshwater‐adapted stickleback) (Figure 1a) (Bourgeois et al., 1994; Campbell, 1985; Giles, 1983; Haenel et al., 2019a; Klepaker et al., 2016; Magalhaes et al., 2016; Spence et al., 2013). The function of this evolution is likely to be reduced metabolic demands, conferring an advantage in nutrient‐depleted acidic habitats. (Note that for simplicity, we will use the terms acidic habitats and acidic adaptation throughout this paper, but we acknowledge that selection may not necessarily be mediated by pH [alone], but by an associated shortage in dissolved ions.) Although marine threespine stickleback have colonized innumerable freshwater habitats across the northern hemisphere, morphological adaptation to acidic habitats is reported only from relatively few locations across the species’ range (Campbell, 1985; Bourgeois et al., 1994; Klepaker et al.,^2013^ ). An exception is North Uist (Outer Hebrides, Scotland) (Figure 1b), an island on which acidic‐adapted stickleback ecomorphs are common. Due to its particular surface geology (Waterston et al., 1979), the eastern part of this island harbours numerous acidic lakes (pH around 5–6) inhabited by archetypal acidic‐adapted stickleback that have probably evolved multiple times independently (Giles, 1983; Haenel et al., 2019a; Klepaker et al., 2016; Magalhaes et al., 2016; Spence et al., 2013). This parallel evolution has occurred though the deterministic sorting of SGV available in the marine ancestor, because alleles recruited repeatedly for acidic adaptation are consistently found in extant marine stickleback breeding in coastal habitats of North Uist, albeit generally at modest to low frequency (Haenel et al., 2019a). What remains unknown is whether this SGV primarily reflects the continued flow of acid‐favoured alleles into marine stickleback by hybridization, or whether alleles beneficial to acidic adaptation segregate largely neutrally at these frequencies in marine fish.

**FIGURE 2:**
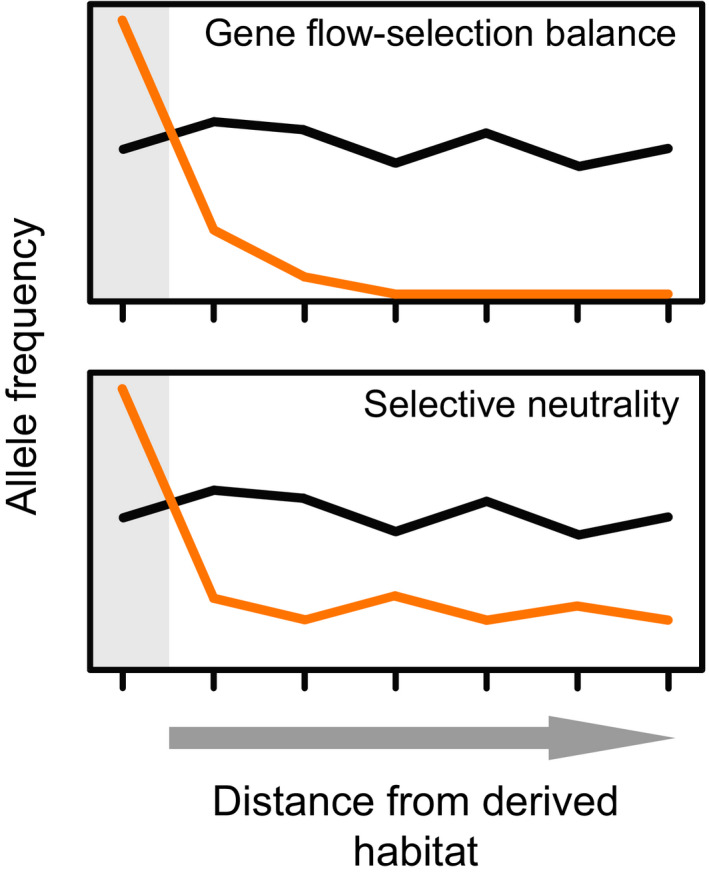
Two alternative explanations for the maintenance of adaptive standing genetic variation (SGV) in ancestral populations. Under gene flow–selection balance (top), genetic variants adaptive and hence at high frequency within a derived habitat (grey background shading) are unconditionally disfavoured in the ancestral habitat (white background shading). These variants, however, may still occur at appreciable frequency in the ancestral habitat if hybridization between populations from the two habitats leads to gene flow. A prediction based on this scenario is that if the opportunity for hybridization is geographically restricted, the frequency in the ancestral habitat of variants favoured in the derived habitat should decline with increasing distance from the derived habitat (orange curve; the ticks represent hypothetical sample sites) because purifying selection increasingly outbalances gene flow. Such spatial change in allele frequencies would not be expected at ecologically neutral polymorphisms (black curve). Under selective neutrality (bottom), we assume that alleles favoured in the derived habitat are selectively neutral within the ancestral habitat when their frequency is relatively low, thus allowing their persistence. The key prediction under this latter scenario is that the frequency in the ancestral habitat of variants favoured in the derived habitat does not decline with increasing geographical distance from the derived habitat

To address this question, we here use whole‐genome sequence data to examine SGV in marine stickleback across the Atlantic Ocean. We hypothesize that if the presence of SGV relevant to acidic adaptation in marine stickleback around North Uist reflects a balance between gene flow and purifying selection, the frequency of alleles favoured in acidic habitats should be elevated in marine stickleback breeding around North Uist compared to marine stickleback sampled from more distant locations (Figure 2, top). The reason is that acidic lakes represent an uncommon freshwater habitat outside North Uist, and the acidic‐adapted ecomorphs common on this island are rare on a worldwide basis. Purifying selection should therefore vastly outbalance the input of deleterious acidic‐favoured alleles by hybridization in marine stickleback far from North Uist. Alternatively, the frequency of acidic‐favoured alleles may not be elevated in marine stickleback breeding around North Uist compared to marine fish in general (Figure 2, bottom), suggesting that purifying selection against these alleles is weak or absent in marine stickleback at large. As we show, our data support this latter scenario, thus highlighting selective neutrality as an underappreciated explanation for the maintenance of SGV.

## 2 MATERIAL AND METHODS

### 2.1 Stickleback samples, DNA library preparation and sequencing

A precondition for our analysis of SGV in marine stickleback was the initial identification of genetic polymorphisms important to acidic adaptation. For this, we considered five acidic and five basic lakes from North Uist from which individual DNA was already available (Haenel et al., 2019a,2019b) (Figure 1b, Table S1). We refer to the latter habitat type as “basic” for terminological consistency with our previous work, but emphasize that the fish inhabiting these lakes represent the standard freshwater stickleback ecomorph widespread across the range of *Gasterosteus aculeatus*. We chose 20 individuals from each of these freshwater populations at random and combined their DNA to equal molarity without PCR (polymerase chain reaction)‐enrichment into either an acidic or a basic pool of 100 individuals each. The goal of this pooling (and the subsequent pooled sequencing, hereafter poolSeq) was to obtain relatively precise allele frequency estimates for acidic versus basic stickleback in general, while ignoring allele frequencies within each specific population. To nevertheless have access to individual genotypes and haplotype information, we additionally chose two individuals from each acidic and basic population at random for individual sequencing (indSeq).

To explore the extent to which adaptive genetic variation discovered in freshwater fish is present as SGV in marine stickleback, we focused on samples from six locations across the Atlantic Ocean: North Uist (NU), Ireland (IR), The Netherlands (NL), Germany (DE), Iceland (IS) and Eastern Canada (CA) (Figure 1b; Table S1; note that North Uist subsumes two nearby marine sample sites, ARDH and OBSM). From each of these marine locations, we aimed for a sample size of around 25 individuals. Except for North Uist, from which marine individual‐level whole‐genome sequence data were already available (Haenel et al., 2019a,2019b), individual DNA was extracted using the Quick‐DNA Miniprep Plus Kit (Zymo Research). For the estimation of population allele frequencies via poolSeq, individual DNA was then combined to equal molarity without PCR‐enrichment within each of the five new locations. In addition, four individuals from each of these locations were chosen at random for indSeq (Table S1).

The 47 total DNA libraries (seven pools and 40 individuals) were paired‐end sequenced to 150 bp together on a single S4 flow cell of an Illumina NovaSeq 6000 instrument, producing a genome‐wide median read depth per base pair of 85× on average across the pools, and of 16× across the individuals (details given in Table S1).

### 2.2 SNP discovery

Raw sequences reads (Haenel et al., 2019b,^2021^) were parsed by library (pool or individual) and aligned to the third‐generation stickleback reference genome assembly (Glazer et al., 2015) by using novoalign (version 4.0, http://www.novocraft.com/products/novoalign/; alignment settings provided in the Supplementary Codes). From the alignments, we derived nucleotide counts (pileups) for all genome‐wide positions by using the *pileup* function from the *Rsamtools* rpackage (Morgan et al., 2017; unless specified otherwise, all analyses were implemented with the rlanguage, version 3.6.0; rDevelopment Core Team, 2019). Single‐nucleotide polymorphisms (SNPs) were then ascertained in two ways: for an initial exploration of population structure among our marine and freshwater samples, we used the pileup data derived from indSeq. Genomic positions qualified as SNPs if the minor allele frequency (MAF) was at least 0.04 across the 24 marine individuals (thus excluding positions appearing variable due to sequencing error only); if cumulative read depth across the marine fish was no greater than 1000 (thus effectively eliminating repeated genomic elements); if all 44 stickleback individuals displayed at least 1× read depth (thus excluding positions with missing data); and if the physical distance to the nearest SNP was at least 100 bp (thus ruling out SNP clusters caused by micro‐indels). This stringent quality filtering resulted in our “indSeq SNPs” including 1.65 million markers across the 447‐Mb stickleback genome. Analyses based on an alternative SNP panel (1.61 million SNPs) obtained by applying the MAF and cumulative read depth threshold to the 20 freshwater instead of the marine individuals consistently produced similar results (details not reported).

For the discovery of genetic variation important to acidic adaptation and the subsequent exploration of SGV, SNPs were ascertained based on the poolSeq data from the acidic and basic fish. We here required a read depth between 100 and 500× and a MAF of at least 0.25 across the two pools combined, and a read depth of at least 50× within each pool. The 1.5 million “poolSeq SNPs” passing these filters were genotyped in all freshwater and marine population pools separately.

### 2.3 Population structure

As a first analytical step, we explored population structure based on genealogies derived from the indSeq SNPs. The purpose was to develop a sense for the genetic relatedness among marine stickleback across the Atlantic Ocean, and to reassess the relatedness of the freshwater populations among each other and to marine fish based on SNP data from whole‐genome indSeq (in Haenel et al., 2019a the latter was done with SNPs derived from pooled RADseq [restriction site‐associated DNA sequencing]). For computational efficiency, we reduced the full indSeq SNP panel to a random subset of 200,000 autosomal SNPs, additionally considering sample sizes of 100,000 and 15,000 SNPs in supplementary analyses (all these data sets were largely independent, as the choice of SNPs was random). For all 44 marine and freshwater individuals, we then derived haploid multilocus genotypes by drawing at each SNP the more frequent allele, or a random allele when both were equally frequent. This haploid strategy (Berner, 2021) circumvented the ambiguity of diploid genotyping in individuals with low read depth. The haploid genotypes were then concatenated to nucleotide strings in fasta format.

The genotype data above were derived from SNPs chosen at random across the genome. However, both marine–freshwater and acidic–basic divergence in stickleback involves selection on numerous loci across the genome (Bassham et al., 2018; Fang et al., 2020; Haenel et al., 2019a; Jones, Grabherr, et al., 2012; Roesti et al., 2014; Terekhanova et al., 2019). To assess to what extent natural selection influences population structure, we additionally explored the genetic relatedness among our marine and freshwater individuals based on a subset of indSeq SNPs filtered to reduce the influence of selection. Following the strategy of Haenel et al. (2019a), we excluded SNPs exhibiting an absolute allele frequency difference (*AFD*; Berner, 2019) >0.4 in both a global marine–freshwater comparison performed by pooling two random nucleotides drawn from the pileup of each individual at each SNP within the marine vs. freshwater group of individuals, and in the acidic–basic comparison described below. As the latter included an MAF threshold of 0.25, we applied the same threshold in the marine–freshwater comparison. Moreover, we here considered exclusively SNPs located within the peripheral 5 Mb of each chromosome (Berner & Roesti, 2017). These regions display particularly high recombination rates in stickleback (Glazer et al., 2015; Roesti et al., 2013), and hence are those least affected by hitchhiking (linked selection). The 120,448 SNPs passing these filters were treated as above to obtain haploid genotype strings. We hereafter call the randomly chosen genotype data “Random SNPs” and the markers chosen to reduce the footprint of selection “Neutral SNPs”, emphasizing that in the latter, a signal of selection may still persist.

For an earlier investigation of the genetic relatedness among North Uist stickleback based on poolSeq data, we used synthetic multilocus genotypes generated by concatenating alleles drawn from RAD‐sequenced sample pools (Haenel et al., 2019a), thereby erasing individual‐level haplotype structure. To assess the value of such synthetic genotypes for capturing genetic structure among populations, we here pooled the nucleotide counts at a number of random and neutral SNPs matching the individual‐level data described above. We then drew a single nucleotide per sample location according to the observed pooled allele frequencies, and saved these draws concatenated to a single haploid nucleotide string per location in fasta format. The synthetic genotype data produced in this way allowed comparing genealogies based on truly individual‐aware vs. synthetic genotypes derived from the same SNP panel.

Based on the genotype files, genealogies were generated by using the *ape* (version 5; Paradis & Schliep, 2018) and *phangorn* (version 2.5.5; Schliep, 2011) r packages. We determined the most appropriate models of sequence evolution (mostly GTR+G), constructed maximum‐likelihood genealogies, and visualized them as unrooted phylograms. Node support was determined based on 500 bootstrap iterations. As an alternative to phylograms, we also considered exploring population structure by ordination (principal coordinates analysis). However, the proportion of variation captured by the first ordination axes was consistently small (~8% or less). We therefore considered ordination an ineffective tool for pattern recognition.

### 2.4 Identifying alleles important to acidic adaptation, and quantifying their frequencies in marine stickleback

To identify alleles important to the adaptation of stickleback to acidic habitats, we performed genome‐wide differentiation mapping between the acidic and basic sample pools. That is, we scanned the poolSeq SNPs for positions exhibiting extremely high global differentiation between stickleback from acidic vs. basic lakes. The reason why we did not define genetic variation important for acidic adaptation simply as SNPs highly differentiated between acidic and marine fish is that this would mostly have uncovered genetic variation important to marine–freshwater divergence in general. Such variation is abundant in North Uist stickleback (Figure S3 in Haenel et al., 2019a; see also Jones, Grabherr, et al., 2012; Roesti et al., 2014; Bassham et al., 2018; Fang et al., 2020; Terekhanova et al., 2019). Our focus, however, was specifically on genetic variation for which gene flow into marine fish must be rare and geographically restricted. Acidic–basic differentiation was expressed by the absolute allele frequency difference *AFD*. Positions qualified as high‐differentiation SNPs if they showed *AFD* ≥ 0.85, approximately corresponding to the top 0.01 percent of the *AFD* distribution. This *AFD* threshold was more stringent than in Haenel et al. (2019a) (0.70) because a higher marker resolution was available, and was chosen to maximize the strength of acidic–basic differentiation while still yielding an adequate number of SNPs for downstream analyses. The positions were further required to be autosomal, and to be physically separated from each other by at least 100 kb to ensure independence (tight linkage disequilibrium typically decays over much shorter distances in stickleback; e.g., Roesti et al., 2015). With these criteria, we obtained a panel of 50 “adaptive SNPs”, that is, positions at which one allele appears strongly and consistently selectively favoured in acidic habitats. As a basis for comparison, we analogously selected a panel of 500 “baseline SNPs” from the same genome scan. These latter polymorphisms were also required to be separated by at least 100 kb, but to exhibit minimal differentiation (*AFD* within 0.1% of the genome‐wide median) between the acidic and the basic pool. The latter criterion ensured that these SNPs did not tag genome regions (consistently) involved in acidic adaptation. At each of the adaptive SNPs, we then defined the nucleotide predominant in the acidic pool as the “acidic allele,” and determined and graphed the frequency of these alleles in all six marine sample pools. An analogous analysis was performed for the baseline SNPs, here defining the acidic allele as the one relatively more common in the acidic than the basic pool. Our prediction was that if genetic variation at the adaptive SNPs in marine stickleback reflects gene flow–selection balance, the frequency of the acidic alleles at these markers (but not at the baseline SNPs) should be elevated in marine stickleback sampled on North Uist. As a resource, we additionally compiled all genes located within a 100‐kb window centred at each adaptive SNP.

For three exemplary adaptive SNPs, we further visualized the diversity and distribution of surrounding haplotypes among our samples based on haplotype networks. The markers chosen included the adaptive SNP exhibiting the strongest acidic–basic differentiation in the present study (*AFD* = 0.96), the adaptive SNP tagging the genome region showing the strongest acidic–basic differentiation in a previous investigation (Figure 3a in Haenel et al., 2019a), and the adaptive SNP located on a known inversion polymorphism (Haenel et al., 2019a; Jones, Grabherr, et al., 2012; Roesti et al., 2015). Using the raw nucleotide counts derived from indSeq, we performed individual diploid genotyping for all nucleotide positions exhibiting a read depth of 10× or greater across a 5‐kb window centred on the adaptive SNPs, considering positions as heterozygous if their MAF was >0.1. Individuals with >25% missing genotypes were omitted. Based on the remaining data, positions qualified as informative SNPs if they displayed ≤40% missing genotypes and a MAF of at least 0.05. The resulting genotype matrices were subjected to phasing with fastphase version 1.4.8 (Scheet & Stephens, 2006; settings provided in the Supplementary Codes). Haplotype genealogies were then constructed with raxml version 8 (Stamatakis, 2014) and visualized as haplotype networks in fitchi (Matschiner, 2016) (settings provided in the Supplementary Codes).

**FIGURE 3:**
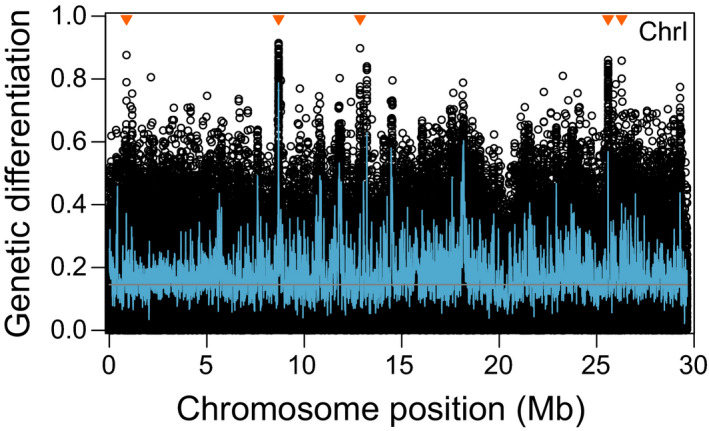
Genetic differentiation, quantified by the absolute allele frequency difference *AFD*, between the acidic and basic stickleback pool along an exemplary chromosome. The black circles represent individual SNPs, the blue curve shows average differentiation across sliding windows of 10 kb with 5‐kb overlap (windows with fewer than six SNPs were discarded), and the grey line gives the genome‐wide median differentiation (0.145). The orange triangles denote the adaptive SNPs on this chromosome; that is, the markers exhibiting extremely strong and consistent acidic–basic differentiation used to explore adaptive standing genetic variation in marine stickleback

## 3 RESULTS AND DISCUSSION

### 3.1 Population structure

Our high‐resolution SNP genealogies revealed consistent yet modest genetic structure among marine stickleback from the Atlantic. Specifically, the phylograms based on SNPs both chosen randomly across the genome and filtered stringently to reduce the influence of selection recovered three marine branches (Figure 1c; bootstrap support is given in Figure S1). These branches were formed by the marine individuals from North Uist and Ireland (ARDH, OBSM, IR), the two samples from the North Sea (DE, NL), and stickleback from Canada and Iceland (CA, IS). Within these branches, however, marine fish from a given location generally did not emerge as monophyletic, except for the Canadian individuals collected thousands of kilometres from the nearest sampling locations (IR, IS) (Figure 1b). In contrast to the marine fish, our freshwater samples exhibited genetic structure differing fundamentally between the random and neutral SNP panels (Figure 1c). Based on the former, all freshwater stickleback together grouped to a single, well‐supported branch distinct from marine fish, and within this freshwater branch, individuals clustered almost perfectly according to acidic vs. basic habitat. This ecological structure largely vanished when using SNPs ascertained to reduce the influence of selection. Moreover, contrary to marine stickleback, freshwater individuals almost consistently grouped by sampling location, despite the dramatically smaller geographical distance among the lakes compared to the marine locations (Figure 1b). All these patterns remained qualitatively consistent when using sparser data sets, and when replacing individual‐level by synthetic genotypes derived from pooled data (Figure S1). The latter confirms that poolSeq data enable meaningful genealogical analyses at the population level (Haenel et al., 2019a).

The modest genetic structure among our marine locations within the three marine branches is consistent with the notion that marine stickleback display large population sizes, and that genetic drift is relatively weak (Catchen et al., 2013; Hohenlohe et al., 2010; Jones, Chan, et al., 2012; Lescak et al., 2015; Mäkinen et al., 2006; Roesti et al., 2014). This view is also well supported by the comparison of genetic differentiation among marine vs. among freshwater samples: while genome‐wide median *AFD* was 0.132 across all pairwise marine sample comparisons (0.019 when expressed by *F*_ST_, Nei, 1973; individual values are presented in Table S2), much higher values were observed across the pairwise comparisons between freshwater populations (*AFD* = 0.219, *F*_ST_ =0.068). (The latter values were derived from differentiation data presented in Table S2 of Haenel et al., 2019a; indSeq performed for the present study included too few individuals per population, and poolSeq used combinations of individuals from multiple populations, both precluding the reliable estimation of population differentiation.) Given weak drift in marine stickleback, we expect that deleterious genetic variation introduced by hybridization with freshwater fish should be eliminated efficiently. Nevertheless, stickleback across the Atlantic clearly do exhibit genetic structure related to geography. Assuming gene flow–selection balance as a cause for the maintenance of SGV, we would therefore expect differences in the level of SGV among broad regions within the Atlantic if these regions differed in the input of maladaptive acidic alleles. A further insight into marine stickleback emerging from both the random and neutral SNPs is that the freshwater populations from North Uist are genetically no more similar to marine fish sampled in immediate (ARDH, OBSM) or relative (IR) proximity than to the samples from the much more distant marine locations. This implies that at the genome‐wide level, any Atlantic marine sample—irrespective of its precise geographical origin (and including offshore samples such as IR; Table S1)—serves as an adequate representation of ancestral Atlantic marine stickleback (see also Kirch et al., 2021).

An intriguing finding emerging from the genealogy is the nearly perfect segregation of stickleback by habitat when using SNPs sampled at random across the genome. At first glance, this may stimulate the interpretation that on North Uist, initially a single freshwater stickleback form evolved, subsequently differentiated into a single acidic and basic ecomorph, and these ecomorphs then split into multiple subpopulations. Apart from being hydrogeographically implausible (see the Supporting Discussion in Haenel et al., 2019a), this interpretation is challenged by the genetic structure revealed by the neutral SNPs: the deep separation of freshwater populations on North Uist based on this marker panel indicates that acidic and basic ecomorphs have arisen multiple times independently through the adaptive sorting of ancestral marine SGV (Magalhaes et al., 2016; Haenel et al., 2019a; see also Bell et al., 1993). The contrasting results obtained from random vs. neutral SNPs in freshwater but not marine stickleback highlight, on the one hand, how deterministically genome‐wide polygenic selection and associated hitchhiking during freshwater adaptation can shape genetic population structure and thus confound neutral evolutionary history (see also Berner, 2021; Berner & Roesti, 2017). On the other hand, these results indicate that the genomes of stickleback populations recently adapted to ecologically novel freshwater habitats are much more profoundly shaped by selection than the genomes of the ancestral marine form. Nevertheless, the deep separation among the freshwater populations observed in both types of genealogies (and mirrored by genome‐wide differentiation; Table S2 in Haenel et al., 2019a) make clear that drift associated with relatively small population size has also played a fundamental role in the evolution of our acidic and basic stickleback populations.

### 3.2 Loci important to acidic adaptation and their allele frequencies across Atlantic stickleback

**FIGURE 4:**
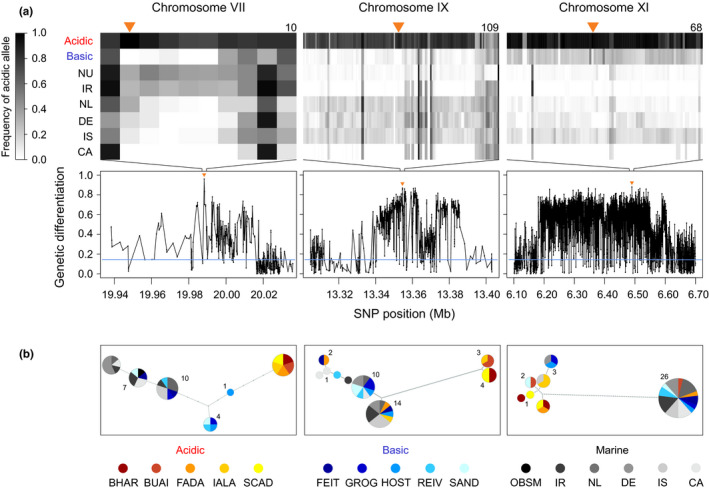
Loci important to acidic adaptation, and their allele frequencies and haplotypes across samples. The lower panels in (a) show three exemplary genome regions exhibiting strong differentiation between the acidic and basic stickleback pools. The dots connected by lines represent individual SNPs, and the horizontal blue line indicates genome‐wide median differentiation. The markers exhibiting the highest differentiation in these regions are marked by orange triangles and were included in the panel of adaptive SNPs (*AFD *≥ 0.85). The adaptive SNP on chromosome VII is the most strongly differentiated marker in our study, while the locus on chromosome IX showed the strongest acidic–basic differentiation in a previous genome scan (Figure 3A in Haenel et al., 2019a). The locus on chromosome XI is an inversion. The width of the visualized chromosome window is 100 kb for the loci on chromosomes VII and IX, and 600 kb for the inversion locus. The upper panels in (a) indicate for each freshwater and marine stickleback pool the frequency of the allele predominant in the acidic pool (acidic allele) at all SNPs within a 5‐kb window centred at the three adaptive SNPs. Each SNP is a separate column, and the number of SNPs is indicated on the top right of each panel. The NU pool combines marine individuals from the North Uist sites ARDH and OBSM. (b) Haplotype genealogies based on phased genotypes derived from individual sequencing at SNPs across the same 5‐kb windows. Pies represent unique haplotypes and edges connecting pies or nodes indicate one inferred mutational step. Within each panel, sample size is given for one pie per size class. Note that the acidic populations generally share haplotypes highly distinct from those prevailing in the marine samples and in the basic populations

Our analysis of genetic structure revealed striking genome‐wide evidence of selection, including between acidic and basic ecomorphs. To investigate how polymorphisms important to acidic adaptation are maintained as SGV in marine stickleback, we searched for loci consistently involved in acidic adaptation based on the genome‐wide comparison of acidic vs. basic poolSeq data (Figure 3; differentiation profiles across all chromosomes are presented in Figure S2). This identified 50 independent adaptive SNPs nearly fixed for alternative alleles between the two freshwater ecomorphs (*AFD* 0.851–0.960; genome‐wide median differentiation was 0.145) (Figure 4a; all adaptive SNPs are characterized in Table S3, and associated genes listed in Table S4). These adaptive SNPs recovered many of the genome regions identified as important to acidic–basic differentiation in Haenel et al. (2019a), based on partly independent specimen panels and a different analytical approach. Specifically, 15 of the 19 regions of highest acidic–basic differentiation inferred in Haenel et al., 2019a (i.e., the regions containing the “top core SNPs” in that study) also exhibited a marker qualifying as adaptive SNP in the present investigation (Figure 4a; Figure S3). However, given the much higher (whole‐genome) marker resolution, the present study also identified numerous novel regions (Figure 4a; Figure S2). Haplotype networks derived from genotypes phased across 5 kb around three exemplary adaptive SNPs indicated that these markers generally represent longer DNA tracts differentiated between the ecomorphs (Figure 4b). Across these exemplary regions, acidic stickleback populations generally shared closely related haplotypes distinct from the haplotypes prevailing in marine (and basic) fish, although sometimes acidic individuals exhibited marine haplotypes (chromosome IX and XI) and vice versa (chromosome XI).

**FIGURE 5:**
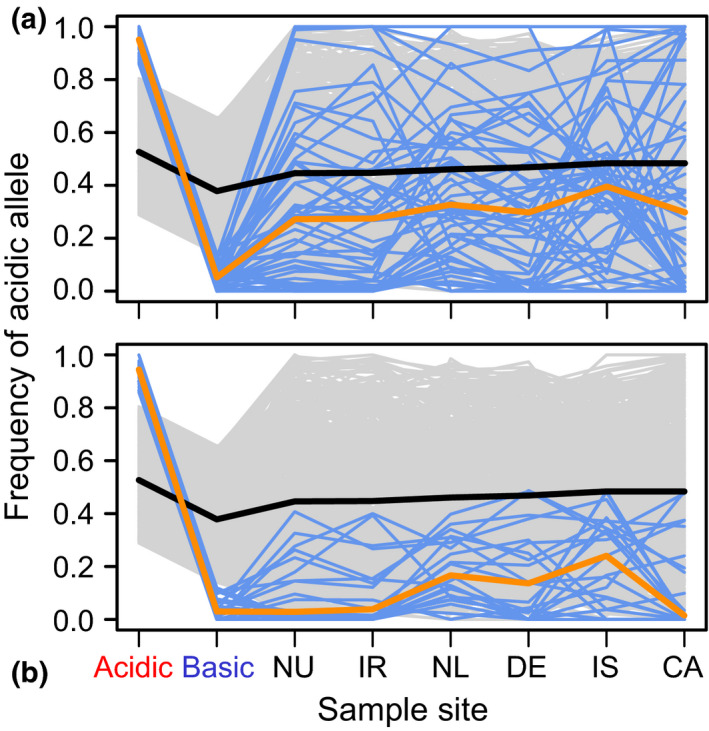
Frequency of the acidic allele at the adaptive and baseline SNPs. (a) The blue lines give the frequency of the acidic allele at each of the 50 adaptive SNPs in each sample pool, and the orange line indicates the median frequency. The grey lines show the acidic allele frequency at 500 baseline SNPs exhibiting a magnitude of acidic–basic differentiation near the genome‐wide median (their median frequency is indicated by the black line). The first two sites from the left are the freshwater pools from North Uist used to identify the adaptive SNPs. The other locations represent marine stickleback (NU combines individuals from the marine North Uist samples ARDH and OBSM). The marine locations are ordered by increasing approximate swimming distance from North Uist. Note that the subtle allele frequency differentiation between the acidic and basic pool at the baseline SNPs is expected technically because at these markers too, the acidic allele was defined as the one relatively more frequent in the acidic than the basic pool. Panel (b) follows the same format as (a) but shows data only for the subset of adaptive SNPs at which the acidic allele is the minor allele within all marine sample pools. Both graphs convey that the frequency of alleles important to acidic adaptation is not elevated in marine stickleback close to North Uist than further away

At the adaptive SNPs, marine stickleback generally exhibited lower frequencies for the alleles characteristic of acidic fish (acidic alleles; median frequency across all SNP by marine sample combinations: 0.30) than for the alleles typical of the basic populations (median frequency 0.70) (Figure 5a; Table S3). Also, the acidic alleles occurred at a lower overall frequency at the adaptive SNPs than at the baseline SNPs not under consistent acidic–basic divergence (median frequency across all baseline SNPs by marine sample combinations: 0.46). A few adaptive SNPs, however, were exceptional in that the acidic allele occurred at consistently high frequency, or even close to fixation, in the ocean (e.g., the SNPs 8, 10 and 28 in Table S3; an exemplary haplotype network for such a SNP is shown in Figure S4). These polymorphisms thus made it into our panel of adaptive SNPs because of massive allele frequency shifts during the adaptation to the basic but not to the acidic habitats.

Overall, these findings are in line with observations in Haenel et al. (2019a) and indicate that alleles presumably important for the adaptation to ecologically highly derived acidic habitats tend to be unfavourable in ancestral marine stickleback when occurring at high frequency. Interestingly, however, we found no indication that the frequency of the acidic allele at the adaptive SNPs was elevated in marine samples collected around North Uist compared to samples from more distant locations (Figure 5a; compatibility intervals for the median frequency of the acidic alleles for all samples are presented in Figure S5); the frequency of these alleles was highly stable across all our marine samples. This key finding was reproduced when considering exclusively the subset of adaptive SNPs at which the acidic allele proved the minor allele within *all* marine samples (*n* = 21; indicated in Table S3) (Figure 5b; Figure S5; median frequency across all SNPs by marine sample combinations: 0.10); that is, the subset of markers at which purifying selection in marine stickleback appears particularly plausible because acidic adaptation involves a particularly strong shift away from the ancestral allele frequency.

The finding of similar frequencies of alleles important to adaptation to acidic waters across Atlantic marine stickleback challenges perpetual antagonism between gene flow and purifying selection (Bassham et al., 2018; Galloway et al., 2020; Schluter & Conte, 2009) as a sufficient explanation for the maintenance of adaptive SGV in the ocean. Instead, we propose that acidic alleles can persist neutrally in marine populations when occurring at moderate to low frequencies. Purifying selection certainly plays a role, but primarily by impeding these alleles from rising to high frequency in marine stickleback. Note that the average frequency of the acidic alleles in the ocean was still around 0.3 (Figure 5a; Figure S5); at many adaptive loci, a substantial proportion of marine stickleback are thus expected to be homozygous for the acidic allele, so that purifying selection should still be effective even when these alleles were recessive. We therefore argue that the reason for the persistence of acidic alleles in marine populations is not their recessivity, but their selective neutrality when relatively uncommon. This interpretation supports quantitative genetic models under which polygenic adaptation can be achieved by moderate allele frequency shifts (Kremer & Le Corre, 2012; Latta, 1998; Le Corre & Kremer, 2012).

An important caveat to consider is that although acidic habitats and the associated stickleback ecomorphs (Figure 1a) are exceptionally common on North Uist and rare elsewhere (Campbell, 1985; Bourgeois et al., 1994; Klepaker et al. 2013), the potential of marine stickleback to hybridize with acidic‐adapted freshwater populations was not explicitly manipulated or controlled among our Atlantic marine samples. Is it plausible that gene flow from acidic‐adapted to marine stickleback is more widespread than we assume, sufficiently so to raise acidic alleles to substantial frequencies in marine stickleback all across the Atlantic despite purifying selection? In our view, the marine samples from the North Sea (DE, NL) refute this concern: western mainland Europe is densely populated and its Ichthyofauna is well investigated, but acidic stickleback ecomorphs have to our knowledge not been reported. Gene flow of acidic alleles into marine fish thus appears highly unlikely across this region, and yet the frequencies of acidic alleles are not reduced in these specific marine samples (Figure 5; Figure S5), consistent with the selective neutrality of these alleles when occurring at the frequencies observed in marine fish. Similar reasoning applies to marine stickleback around Iceland, because highly acidic freshwater habitats seem to be absent in Iceland (Magalhaes et al., 2021).

## 4 CONCLUSIONS

Adaptation commonly occurs from standing genetic variation, but how this variation is maintained in ancestral populations is little explored. We have here presented observational evidence suggesting that, overall, genetic variants important to adaptation to a highly derived habitat are maintained at moderate frequencies within the ancestral habitat. These variants do not appear to occur in higher frequencies in geographical regions where ancestral populations have a higher opportunity for gene flow from derived populations. We thus conclude that long‐term gene flow–selection balance is an incomplete explanation for the maintenance of SGV. Instead, we propose that purifying selection of these variants in the ancestral habitat subsides as their frequency decreases, thus allowing their neutral persistence. This novel perspective on the maintenance of SGV should now be scrutinized by controlled experimental work quantifying the fitness consequences of individual genetic variants across different habitats and genomic backgrounds.

## CONFLICT OF INTEREST

The authors declare no competing interests.

## AUTHOR CONTRIBUTIONS

D.B. and Q.H. conceived the study; A.M. provided all freshwater and marine samples from North Uist; Q.H. performed wet laboratory work; Q.H., D.B. and L.G. wrote code and analysed genomic data; Q.H. and D.B. interpreted the results and wrote the manuscript.

## Supporting information

Supplementary Material

Supplementary Material

## Data Availability

Raw Illumina sequences for all individuals and pools are available from the NCBI Sequence Read Archive under BioProject no. PRJNA485717 (indSeq data from ARDH and OBSM), and from the European Nucleotide Archive under project no. PRJEB42736 (all other data). All code used for data analysis is provided as Supplementary Code in the Supporting Information.
