# Supplementary material for: The maintenance of standing genetic variation: Gene flow vs. selective neutrality in Atlantic stickleback fish
Source: Mol Ecol. 2021 Nov 25;31(3):811–21. doi: 10.1111/mec.16269 (PMC9299253; doi:10.1111/mec.16269)
Supplement: Supplementary file 1 — Supplementary Material [file MEC-31-811-s001.pdf]

MOLECULAR ECOLOGY

Supplemental Information for:

The maintenance of standing genetic variation: gene flow versus selective neutrality in Atlantic stickleback fish

Quiterie Haenel, Laurent Guerard, Andrew D. C. MacColl and Daniel Berner

Table of Contents:

**TABLE S1**	**Description of the samples**	Page 2
**TABLE S2**	**Pairwise genetic differentiation among the marine samples**	Page 3
**TABLE S3**	**Detailed characterization of the 50 adaptive SNPs**	Page 4
**TABLE S4**	**Candidate genes around the 50 adaptive SNPs**	Page 5-11
**FIGURE S1**	**Phylograms with bootstrap support, and based on smaller marker subsets and synthetic genotypes**	Page 12-13
**FIGURE S2**	**Genetic differentiation between the acidic and basic pool along all chromosomes**	Page 14-16
**FIGURE S3**	**Consistency between the present study and Haenel et al. 2019 in the identification of genome regions important to acidic adaptation**	Page 17-20
**FIGURE S4**	**Haplotype network for an adaptive SNP at which the acidic allele is the common allele in marine stickleback**	Page 21
**FIGURE S5**	**Bootstrap compatibility intervals for the median frequency of the acidic alleles in all samples**	Page 22

TABLE S1 Description of the individual and pooled samples used for the study.

Sample name in study	Original sample name	Habitat	Country	Region	Locality	Latitude	Longitude	Sampling method	Read depth (genome-wide median read depth per base position)	DNA library type	Number of individuals pooled	Comments
BHAR 1	BH4 (Ga2935)	Freshwater, acidic	UK (Scotland)	North Uist	a' Bharpa			Minnow traps	15	individual		Geographic location given in Table 1 of Magalhaes et al. 2016, Molecular Ecology
BHAR 2	BH25 (Ga2956)	Freshwater, acidic	UK (Scotland)	North Uist	a' Bharpa			Minnow traps	18	individual		Geographic location given in Table 1 of Magalhaes et al. 2016, Molecular Ecology
BUAI 1	BU15 (Ga3031)	Freshwater, acidic	UK (Scotland)	North Uist	na Buaile			Minnow traps	18	individual		Geographic location given in Table 1 of Magalhaes et al. 2016, Molecular Ecology
BUAI 2	BU18 (Ga3034)	Freshwater, acidic	UK (Scotland)	North Uist	na Buaile			Minnow traps	24	individual		Geographic location given in Table 1 of Magalhaes et al. 2016, Molecular Ecology
FADA 1	FA5 (Ga3090)	Freshwater, acidic	UK (Scotland)	North Uist	Fada			Minnow traps	19	individual		Geographic location given in Table 1 of Magalhaes et al. 2016, Molecular Ecology
FADA 2	FA22 (Ga3107)	Freshwater, acidic	UK (Scotland)	North Uist	Fada			Minnow traps	17	individual		Geographic location given in Table 1 of Magalhaes et al. 2016, Molecular Ecology
IALA 1	IA7 (Ga3066)	Freshwater, acidic	UK (Scotland)	North Uist	Ialaidh			Minnow traps	22	individual		Geographic location given in Table 1 of Magalhaes et al. 2016, Molecular Ecology
IALA 2	IA24 (Ga3083)	Freshwater, acidic	UK (Scotland)	North Uist	Ialaidh			Minnow traps	20	individual		Geographic location given in Table 1 of Magalhaes et al. 2016, Molecular Ecology
SCAD 1	SC27 (Ga2991)	Freshwater, acidic	UK (Scotland)	North Uist	Scadavay			Minnow traps	19	individual		Geographic location given in Table 1 of Magalhaes et al. 2016, Molecular Ecology
SCAD 2	SC28 (Ga2992)	Freshwater, acidic	UK (Scotland)	North Uist	Scadavay			Minnow traps	16	individual		Geographic location given in Table 1 of Magalhaes et al. 2016, Molecular Ecology
Acidic pool	A-NU_pool	Freshwater, acidic	UK (Scotland)	North Uist				Minnow traps	158	pooled	100	Combines DNA from all five acidic lakes (20 individuals from each)
FEIT 1	FE8 (Ga2891)	Freshwater, basic	UK (Scotland)	North Uist	nam Feithean			Minnow traps	22	individual		Geographic location given in Table 1 of Magalhaes et al. 2016, Molecular Ecology
FEIT 2	FE17 (Ga2900)	Freshwater, basic	UK (Scotland)	North Uist	nam Feithean			Minnow traps	24	individual		Geographic location given in Table 1 of Magalhaes et al. 2016, Molecular Ecology
GROG 1	GR14 (Ga2855)	Freshwater, basic	UK (Scotland)	North Uist	Grogary			Minnow traps	24	individual		Geographic location given in Table 1 of Magalhaes et al. 2016, Molecular Ecology
GROG 2	GR18 (Ga2859)	Freshwater, basic	UK (Scotland)	North Uist	Grogary			Minnow traps	36	individual		Geographic location given in Table 1 of Magalhaes et al. 2016, Molecular Ecology
HOST 1	HO5 (Ga2808)	Freshwater, basic	UK (Scotland)	North Uist	Hosta			Minnow traps	24	individual		Geographic location given in Table 1 of Magalhaes et al. 2016, Molecular Ecology
HOST 2	HO14 (Ga2817)	Freshwater, basic	UK (Scotland)	North Uist	Hosta			Minnow traps	22	individual		Geographic location given in Table 1 of Magalhaes et al. 2016, Molecular Ecology
REIV 1	RE8 (Ga3116)	Freshwater, basic	UK (Scotland)	North Uist	na Reival			Minnow traps	31	individual		Geographic location given in Table 1 of Magalhaes et al. 2016, Molecular Ecology
REIV 2	RE12 (Ga3124)	Freshwater, basic	UK (Scotland)	North Uist	na Reival			Minnow traps	26	individual		Geographic location given in Table 1 of Magalhaes et al. 2016, Molecular Ecology
SAND 1	SA5 (Ga2773)	Freshwater, basic	UK (Scotland)	North Uist	Sandary			Minnow traps	16	individual		Geographic location given in Table 1 of Magalhaes et al. 2016, Molecular Ecology
SAND 2	SA8 (Ga2776)	Freshwater, basic	UK (Scotland)	North Uist	Sandary			Minnow traps	17	individual		Geographic location given in Table 1 of Magalhaes et al. 2016, Molecular Ecology
Basic pool	B-NU_pool	Freshwater, basic	UK (Scotland)	North Uist				Minnow traps	66	pooled	100	Combines DNA from all five basic lakes (20 individuals from each)
ARDH 1	ARDH_1	Marine	UK (Scotland)	North Atlantic	North Uist, tidal lagoon	57°34'44.4"N	7°24'57.6"W	Minnow traps	4	individual		Sequenced individually for Haenel et al. 2019, Evolution Letters. Raw data available from NCBI Sequence Read Archive under BioProject number PRJNA485717
ARDH 2	ARDH_2	Marine	UK (Scotland)	North Atlantic	North Uist, tidal lagoon	57°34'44.4"N	7°24'57.6"W	Minnow traps	5	individual		Sequenced individually for Haenel et al. 2019, Evolution Letters. Raw data available from NCBI Sequence Read Archive under BioProject number PRJNA485717
OBSM 1	OBSM_346	Marine	UK (Scotland)	North Atlantic	North Uist, tidal lagoon	57°36'00.0"N	7°10'00.0"W	Minnow traps	6	individual		Sequenced individually for Haenel et al. 2019, Evolution Letters. Raw data available from NCBI Sequence Read Archive under BioProject number PRJNA485717
OBSM 2	OBSM_716	Marine	UK (Scotland)	North Atlantic	North Uist, tidal lagoon	57°36'00.0"N	7°10'00.0"W	Minnow traps	6	individual		Sequenced individually for Haenel et al. 2019, Evolution Letters. Raw data available from NCBI Sequence Read Archive under BioProject number PRJNA485717
NU marine pool	M-NU_pool	Marine	UK (Scotland)	North Atlantic	North Uist, tidal lagoon			Minnow traps	139	pooled	30	For this pool, genotype data from 10 ARDH and 20 OBSM individuals sequenced individually for Haenel et al. 2019, Evolution Letters, were combined bioinformatically. Raw data from the underlying individuals are available from NCBI Sequence Read Archive under BioProject number PRJNA485717
IR 1	NAO-IRL-11	Marine	Ireland	North Atlantic	North Atlantic, off the Irish coast	56°11'24.0"N	8°59'24.0"W	Research vessel	11	individual		Part of the sample taken at 'Site 1' in Ravinet et al. 2014, Ecology and Evolution
IR 2	NAO-IRL-17	Marine	Ireland	North Atlantic	North Atlantic, off the Irish coast	56°11'24.0"N	8°59'24.0"W	Research vessel	9	individual		Part of the sample taken at 'Site 1' in Ravinet et al. 2014, Ecology and Evolution
IR 3	NAO-IRL-21	Marine	Ireland	North Atlantic	North Atlantic, off the Irish coast	56°11'24.0"N	8°59'24.0"W	Research vessel	10	individual		Part of the sample taken at 'Site 1' in Ravinet et al. 2014, Ecology and Evolution
IR 4	NAO-IRL-23	Marine	Ireland	North Atlantic	North Atlantic, off the Irish coast	56°11'24.0"N	8°59'24.0"W	Research vessel	10	individual		Part of the sample taken at 'Site 1' in Ravinet et al. 2014, Ecology and Evolution
IR pool	NAO-IRL_pool	Marine	Ireland	North Atlantic	North Atlantic, off the Irish coast	56°11'24.0"N	8°59'24.0"W	Research vessel	79	pooled	21	Part of the sample taken at 'Site 1' in Ravinet et al. 2014, Ecology and Evolution
NL 1	BWS-NL-4	Marine	The Netherlands	North Sea	Borsselle, Western Scheldt estuary	51°23'39.9"N	3°52'15.0"E	Minnow traps	8	individual		
NL 2	BWS-NL-5	Marine	The Netherlands	North Sea	Borsselle, Western Scheldt estuary	51°23'39.9"N	3°52'15.0"E	Minnow traps	10	individual		
NL 3	BWS-NL-6	Marine	The Netherlands	North Sea	Borsselle, Western Scheldt estuary	51°23'39.9"N	3°52'15.0"E	Minnow traps	20	individual		
NL 4	BWS-NL-20	Marine	The Netherlands	North Sea	Borsselle, Western Scheldt estuary	51°23'39.9"N	3°52'15.0"E	Minnow traps	6	individual		
NL pool	BWS-NL_pool	Marine	The Netherlands	North Sea	Borsselle, Western Scheldt estuary	51°23'39.9"N	3°52'15.0"E	Minnow traps	89	pooled	28	
DE 1	SYLT-D-f5	Marine	Germany	North Sea	Sylt	55°01'14.6"N	8°26'29.2"E	Minnow traps	10	individual		
DE 2	SYLT-D-f9	Marine	Germany	North Sea	Sylt	55°01'14.6"N	8°26'29.2"E	Minnow traps	13	individual		
DE 3	SYLT-D-m3	Marine	Germany	North Sea	Sylt	55°01'14.6"N	8°26'29.2"E	Minnow traps	15	individual		
DE 4	SYLT-D-m5	Marine	Germany	North Sea	Sylt	55°01'14.6"N	8°26'29.2"E	Minnow traps	13	individual		
DE pool	SYLT-D_pool	Marine	Germany	North Sea	Sylt	55°01'14.6"N	8°26'29.2"E	Minnow traps	41	pooled	26	
IS 1	LL-ICE-538	Marine	Iceland	Greenland Sea	Lónsós Lón	66°07'06.6"N	16°56'51.4"W	Minnow traps	10	individual		
IS 2	LL-ICE-545	Marine	Iceland	Greenland Sea	Lónsós Lón	66°07'06.6"N	16°56'51.4"W	Minnow traps	11	individual		
IS 3	LL-ICE-551	Marine	Iceland	Greenland Sea	Lónsós Lón	66°07'06.6"N	16°56'51.4"W	Minnow traps	20	individual		
IS 4	LL-ICE-810	Marine	Iceland	Greenland Sea	Lónsós Lón	66°07'06.6"N	16°56'51.4"W	Minnow traps	10	individual		
IS pool	LL-ICE_pool	Marine	Iceland	Greenland Sea	Lónsós Lón	66°07'06.6"N	16°56'51.4"W	Minnow traps	94	pooled	25	
CA 1	IV-CA-517	Marine	Canada	North Western Atlantic, Saint Lawrence Gulf	Baie de l'Isle Verte	48°02'55.9"N	69°21'20.7"W	Minnow traps	11	individual		
CA 2	IV-CA-518	Marine	Canada	North Western Atlantic, Saint Lawrence Gulf	Baie de l'Isle Verte	48°02'55.9"N	69°21'20.7"W	Minnow traps	8	individual		
CA 3	IV-CA-529	Marine	Canada	North Western Atlantic, Saint Lawrence Gulf	Baie de l'Isle Verte	48°02'55.9"N	69°21'20.7"W	Minnow traps	10	individual		
CA 4	IV-CA-530	Marine	Canada	North Western Atlantic, Saint Lawrence Gulf	Baie de l'Isle Verte	48°02'55.9"N	69°21'20.7"W	Minnow traps	21	individual		
CA pool	IV-CA_pool	Marine	Canada	North Western Atlantic, Saint Lawrence Gulf	Baie de l'Isle Verte	48°02'55.9"N	69°21'20.7"W	Minnow traps	72	pooled	24	

TABLE S2 Genome-wide median (upper-right semimatrix) and mean (lower-left semimatrix) genetic differentiation, expressed as absolute allele frequency differentiation (AFD), and as F_{ST} (Nei's 1973 estimator G_{ST}) in parentheses, for all pairwise comparisons of marine sample pools. The underlying markers are the 1.5 million poolSeq SNPs ascertained based on the comparison of the acidic versus basic sample pool (sex chromosome and unanchored scaffolds included; ascertaining SNPs directly in the specific marine pool pairs produced very similar differentiation values). For each marine sample comparison, the SNPs were filtered for coverage (total base count across the two alleles >= 50 within each pool) and MAF (>= 0.25 across the two pools combined). Note that computing differentiation for comparisons involving acidic or basic stickleback was not meaningful, as these pools represented mixes of DNA from different replicate populations within each habitat type. However, differentiation among the freshwater populations, and between each freshwater population and North Uist marine stickleback, is described in Table S2 of Haenel et al. 2019.

	**NU**	**IR**	**NL**	**DE**	**IS**	**CA**
**NU**		0.095 (0.010)	0.123 (0.016)	0.123 (0.017)	0.134 (0.019)	0.206 (0.046)
**IR**	0.112 (0.022)		0.132 (0.019)	0.124 (0.017)	0.139 (0.021)	0.201 (0.044)
**NL**	0.148 (0.039)	0.161 (0.046)		0.082 (0.007)	0.123 (0.017)	0.190 (0.039)
**DE**	0.146 (0.037)	0.152 (0.041)	0.099 (0.017)		0.117 (0.015)	0.164 (0.029)
**IS**	0.159 (0.044)	0.169 (0.050)	0.151 (0.040)	0.145 (0.037)		0.156 (0.027)
**CA**	0.234 (0.092)	0.238 (0.096)	0.225 (0.087)	0.204 (0.074)	0.190 (0.063)	

TABLE S3 Characterization of the 50 adaptive SNPs, ordered by chromosome and position. The alleles give the type of polymorphism at each SNP, with the first and second nucleotide representing the allele predominant in the acidic and basic stickleback pool. The AFD values indicate the allele frequency difference between these pools. At the SNPs shaded blue, the acidic allele is the minor allele (frequency < 0.5) in all six (dark) or in at least four (light) of the marine stickleback samples. At the SNPs shaded red, the acidic allele is the major allele (frequency >= 0.5) in all six (dark) or in at least four (light) of the marine samples. The three SNPs printed in bold are the ones visualized in Figure 4.

SNP	Chr	Pos	Alleles (acidic/ basic)	acidic-basic AFD	Frequency of acidic allele in acidic pool	Frequency of acidic allele in basic pool	Frequency of acidic allele in the marine samples					
							NU	IR	NL	DE	IS	CA
1	I	879'044	C/A	0.876	0.992	0.116	0.755	0.790	0.545	0.750	0.829	1.000
2	I	8'680'470	A/C	0.914	0.944	0.030	0.146	0.155	0.340	0.225	0.375	0.178
3	I	12'858'811	T/C	0.898	0.960	0.063	0.636	0.750	0.281	0.222	0.769	0.000
4	I	25'581'749	T/A	0.860	0.967	0.110	0.145	0.121	0.398	0.484	0.322	0.476
5	I	26'278'312	C/T	0.858	0.977	0.119	0.000	0.000	0.115	0.033	0.000	0.000
6	II	4'524'607	T/C	0.853	0.945	0.093	0.454	0.333	0.492	0.286	0.796	0.781
7	II	13'793'172	T/A	0.858	0.036	0.894	0.000	0.000	0.091	0.000	0.000	0.000
8	IV	12'021'899	C/T	0.947	1.000	0.053	0.993	1.000	1.000	1.000	1.000	1.000
9	IV	12'445'286	C/A	0.903	0.955	0.052	0.951	0.911	0.762	0.587	0.872	0.873
10	IV	12'604'827	A/T	0.887	0.950	0.063	0.993	1.000	0.933	0.833	0.990	0.971
11	IV	13'957'151	T/C	0.882	1.000	0.118	0.006	0.000	0.058	0.000	0.037	0.000
12	IV	19'997'604	T/C	0.924	0.955	0.031	0.088	0.042	0.667	0.700	0.406	0.000
13	IV	20'115'769	C/T	0.872	1.000	0.128	1.000	1.000	0.543	0.524	0.067	1.000
14	IV	20'348'982	G/A	0.894	0.000	0.894	0.410	0.462	0.581	0.417	0.455	1.000
15	IV	21'780'217	A/G	0.870	0.941	0.071	0.301	0.462	0.670	0.750	0.414	0.017
16	IV	26'641'810	G/A	0.924	0.974	0.050	0.017	0.000	0.216	0.205	0.326	0.000
17	IV	33'864'589	A/G	0.854	0.977	0.123	0.489	0.455	0.504	0.295	0.108	0.573
18	V	3'953'806	C/G	0.875	0.875	0.000	0.700	0.577	0.538	0.537	0.295	0.443
19	V	8'733'705	T/C	0.870	0.949	0.079	0.556	0.407	0.298	0.267	0.417	0.140
20	V	12'751'826	C/T	0.885	1.000	0.115	0.131	0.271	0.424	0.207	0.143	0.717
21	VII	13'825'503	C/T	0.887	0.887	0.000	0.306	0.186	0.271	0.069	0.524	0.051
**22**	VII	19'986'348	A/G	0.960	0.960	0.000	0.326	0.278	0.310	0.132	0.084	0.000
23	VII	23'508'447	C/T	0.859	0.859	0.000	0.026	0.015	0.146	0.289	0.301	0.375
24	VIII	1'103'291	T/G	0.936	0.935	0.000	0.712	0.643	0.000	0.000	0.000	0.057
25	VIII	7'206'638	T/C	0.868	0.056	0.923	0.029	0.000	0.125	0.000	0.385	0.000
26	IX	7'878'428	G/T	0.879	0.918	0.040	0.117	0.039	0.213	0.095	1.000	0.000
27	IX	12'612'422	G/A	0.872	0.897	0.025	0.115	0.082	0.451	0.317	0.714	0.345
28	IX	13'200'359	T/C	0.936	0.989	0.053	0.988	1.000	0.842	0.909	0.930	1.000
**29**	IX	13'354'585	C/A	0.867	0.867	0.000	0.022	0.000	0.210	0.136	0.160	0.000
30	X	4'259'221	G/A	0.946	0.961	0.015	0.048	0.022	0.036	0.000	0.802	0.968
31	X	10'097'654	T/A	0.877	0.890	0.013	0.096	0.040	0.247	0.300	0.038	0.099
32	X	10'555'758	G/T	0.952	0.952	0.000	0.277	0.400	0.269	0.487	0.344	0.195
33	X	15'307'031	C/T	0.852	0.948	0.097	0.234	0.136	0.360	0.394	0.371	0.351
**34**	XI	6'489'914	T/C	0.878	0.946	0.067	0.026	0.038	0.023	0.000	0.080	0.000
35	XII	5'123'854	G/T	0.901	0.919	0.018	0.484	0.296	0.600	0.542	0.425	0.274
36	XII	6'754'066	C/A	0.864	0.864	0.000	0.000	0.000	0.167	0.000	0.167	0.240
37	XII	17'793'998	T/G	0.929	1.000	0.071	0.500	0.714	1.000	1.000	0.417	0.783
38	XIII	4'342'266	T/A	0.938	1.000	0.063	0.571	0.856	0.173	0.387	0.561	0.103
39	XIII	14'003'722	T/C	0.854	0.995	0.141	0.268	0.527	0.560	0.673	0.442	0.382
40	XIII	18'701'974	A/G	0.865	0.923	0.058	0.263	0.152	0.128	0.000	0.240	0.373
41	XIV	7'260'047	A/G	0.884	0.920	0.036	0.318	0.317	0.652	0.400	0.523	0.322
42	XV	5'954'154	T/A	0.912	0.961	0.048	0.596	0.484	0.862	0.643	0.500	0.585
43	XV	16'209'873	A/G	0.957	0.957	0.000	0.024	0.000	0.314	0.250	0.000	0.000
44	XVI	4'797'943	A/C	0.866	0.866	0.000	0.075	0.073	0.000	0.043	0.120	0.484
45	XVI	6'828'403	G/T	0.881	0.915	0.034	0.407	0.267	0.291	0.380	0.453	0.014
46	XVII	6'781'353	C/A	0.914	0.984	0.067	0.493	0.288	0.648	0.538	0.734	0.608
47	XX	7'959'446	T/A	0.852	0.063	0.915	0.195	0.088	0.556	0.714	0.375	0.636
48	XX	8'597'535	C/T	0.851	0.879	0.027	0.179	0.394	0.084	0.065	0.341	0.034
49	XX	10'599'415	A/G	0.872	0.901	0.029	0.000	0.012	0.074	0.026	0.483	0.000
50	XXI	16'133'430	C/A	0.941	0.962	0.021	0.286	0.290	0.480	0.343	0.457	0.955

TABLE S4 Compilation of the genes located within a 100 kb window around each of the 50 adaptive SNPs, ordered by chromosome and position.

SNP	Chromosome, Position	Gene ID	Gene name
1	chrI 879044	ENSGACG00000004922	ENSGACG00000004922
		ENSGACG00000004927	ENSGACG00000004927
		ENSGACG00000004929	triap1
		ENSGACG00000004934	supt5h
		ENSGACG00000004963	cox7a1
		ENSGACG00000004964	nf1b
		ENSGACG00000004992	SMCO4
2	chrI 868047	ENSGACG00000009072	grik4
3	chrI 12858811	ENSGACG00000011223	ENSGACG00000011223
		ENSGACG00000011230	ca4b
		ENSGACG00000022214	ENSGACG00000022214
		ENSGACG00000011249	ENSGACG00000011249
		ENSGACG00000011251	ywhag2
		ENSGACG00000011259	camkk1b
		ENSGACG00000011266	ENSGACG00000011266
		ENSGACG00000011279	ENSGACG00000011279
		ENSGACG00000011281	c2cd3
		ENSGACG00000011291	p4ha3
		ENSGACG00000011312	or129-1
		ENSGACG00000011316	diabloa
		ENSGACG00000011318	ENSGACG00000011318
		ENSGACG00000011320	ENSGACG00000011320
4	chrI 25581749	ENSGACG00000014600	zgc:172122
		ENSGACG00000014601	ENSGACG00000014601
		ENSGACG00000014605	u2af1
		ENSGACG00000014627	cbsa
		ENSGACG00000014641	ENSGACG00000014641
		ENSGACG00000014643	ENSGACG00000014643
		ENSGACG00000014645	stoml2
		ENSGACG00000014669	CYP4F8
5	chrI 26278312	ENSGACG00000014299	spega
		ENSGACG00000014313	CTDSP1
		ENSGACG00000021310	MIR26B
		ENSGACG00000014321	ENSGACG00000014321
		ENSGACG00000014323	obsl1a
		ENSGACG00000014324	atp1a1a.2
6	chrII 4524607	ENSGACG00000014582	pepd
7	chrII 13793172	ENSGACG00000014321	ENSGACG00000014321
		ENSGACG00000016060	ddx21
		ENSGACG00000016061	casc1
		ENSGACG00000016064	ENSGACG00000016064
		ENSGACG00000016067	mpc1
		ENSGACG00000016068	kifbp
		ENSGACG00000016070	ENSGACG00000016070
		ENSGACG00000016072	vps26a
		ENSGACG00000016077	supv3l1
		ENSGACG00000016082	hkdc1

8	chrIV 12021899	ENSGACG00000018224	zdhhc15b
		ENSGACG00000018229	uprt
		ENSGACG00000018231	abcb7
9	chrIV 12445286	ENSGACG00000018271	rhogd
		ENSGACG00000018273	ogt.1
		ENSGACG00000022698	ENSGACG00000022698
		ENSGACG00000018279	gcna
		ENSGACG00000018281	cetn2
		ENSGACG00000018285	nsdhl
		ENSGACG00000018286	ENSGACG00000018286
		ENSGACG00000018287	ENSGACG00000018287
		ENSGACG00000018289	fut11
		ENSGACG00000018291	rab9b
		ENSGACG00000018292	plp1a
10	chrIV 12604827	ENSGACG00000018296	nlgn3a
11	chrIV 13957151	ENSGACG00000018422	cpeb4a
		ENSGACG00000018432	stc2a
		ENSGACG00000018433	nkx2.5
		ENSGACG00000018435	bnip1a
		ENSGACG00000018438	atp6v0e1
		ENSGACG00000018439	rpl26
		ENSGACG00000018440	ppp2r2ca
12	chrIV 19997604	ENSGACG00000019554	atoh8
		ENSGACG00000019555	tmem129
		ENSGACG00000019558	rnf103
		ENSGACG00000019560	abhd18
		ENSGACG00000019563	igbp1
		ENSGACG00000019568	magt1
		ENSGACG00000019572	fbxo38
13	chrIV 20115769	ENSGACG00000019538	zgc:113425
		ENSGACG00000019540	rab33ba
		ENSGACG00000019542	hspa9
		ENSGACG00000019553	slitrk2
14	chrIV 20348982	ENSGACG00000019519	slc38a4
		ENSGACG00000019520	slc38a2
		ENSGACG00000019521	ENSGACG00000019521
		ENSGACG00000019522	arid2
		ENSGACG00000022878	ENSGACG00000022878
15	chrIV 21780217	ENSGACG00000019344	mkln1
		ENSGACG00000019341	nfyba
		ENSGACG00000019342	ENSGACG00000019342
		ENSGACG00000019343	ENSGACG00000019343
		ENSGACG00000021429	ENSGACG00000021429
		ENSGACG00000022316	MIR29A
16	chrIV 26641810	ENSGACG00000018964	ENSGACG00000018964
		ENSGACG00000018957	CDPF1
		ENSGACG00000018958	pparaa
		ENSGACG00000022207	ENSGACG00000022207
		ENSGACG00000021259	MIRLET7A3
		ENSGACG00000018960	atxn10
17	chrIV 33864589	ENSGACG00000000636	ncaph2

		ENSGACG00000000639	sco2
		ENSGACG00000000640	ENSGACG00000000640
		ENSGACG00000000642	flncb
		ENSGACG00000000650	kcnd2
		ENSGACG00000000652	tspan12
		ENSGACG00000000654	ing3
		ENSGACG00000000655	ENSGACG00000000655
		ENSGACG00000000657	wnt16
18	chrV 3953806	ENSGACG00000005496	usp22
		ENSGACG00000005506	cops3
		ENSGACG00000005546	NT5M
		ENSGACG00000005572	rasd1
		ENSGACG00000005578	pemt
19	chrV 8733705	ENSGACG00000002892	ENSGACG00000002892
		ENSGACG00000002871	myoz1b
		ENSGACG00000002878	synpo2lb
		ENSGACG00000002883	sec24c
		ENSGACG00000002888	ENSGACG00000002888
		ENSGACG00000002890	ENSGACG00000002890
		ENSGACG00000002901	si:ch73-127m5.1
		ENSGACG00000002906	ENSGACG00000002906
20	chrV 12751826	ENSGACG00000007789	ENSGACG00000007789
		ENSGACG00000007794	si:dkey-32n7.7
		ENSGACG00000007797	vkorc1
		ENSGACG00000007803	mapk7
		ENSGACG00000007820	ENSGACG00000007820
		ENSGACG00000007835	pax10
		ENSGACG00000007839	mmp25b
		ENSGACG00000007849	ca15b
		ENSGACG00000007888	ENSGACG00000007888
		ENSGACG00000007890	ENSGACG00000007890
		ENSGACG00000007899	ENSGACG00000007899
		ENSGACG00000007901	hrc
		ENSGACG00000007920	si:dkey-94f20.4
21	chrVII 13825503	ENSGACG00000020116	ENSGACG00000020116
		ENSGACG00000020117	HSPA8
		ENSGACG00000021746	SNORD14
		ENSGACG00000020118	ENSGACG00000020118
		ENSGACG00000020119	ENSGACG00000020119
		ENSGACG00000020120	bsx
		ENSGACG00000020121	lim2.1
22	chrVII 19986348	ENSGACG00000020345	ENSGACG00000020345
		ENSGACG00000020344	PLS3
		ENSGACG00000020346	ccdc61
		ENSGACG00000020347	itpkca
		ENSGACG00000020348	ENSGACG00000020348
		ENSGACG00000020349	ppm1nb
		ENSGACG00000020350	rtn2b
		ENSGACG00000020351	nectin3b
		ENSGACG00000020352	or133-3
		ENSGACG00000020353	ppme1
		ENSGACG00000020354	ucp2
		ENSGACG00000020355	dnajb13
		ENSGACG00000020356	rab6a

23	chrVII 23508447	ENSGACG00000020590	mtnr1bb
		ENSGACG00000020591	timm10b
		ENSGACG00000020592	ENSGACG00000020592
		ENSGACG00000020593	smpd1
		ENSGACG00000020594	tpte
		ENSGACG00000020595	vps36
24	chrVIII 1103291	ENSGACG00000002165	ENSGACG00000002165
		ENSGACG00000020968	5S_rRNA
		ENSGACG00000021717	5S_rRNA
		ENSGACG00000021183	U1
		ENSGACG00000021620	U5
		ENSGACG00000002169	ENSGACG00000002169
		ENSGACG00000002171	nxpe3
25	chrVIII 7206638	ENSGACG00000006030	ttc14
		ENSGACG00000006044	ENSGACG00000006044
		ENSGACG00000006048	ENSGACG00000006048
		ENSGACG00000006052	fxr1
		ENSGACG00000006076	dnajc19
26	chrIX 7878428	ENSGACG00000018741	gde1
		ENSGACG00000018744	si:dkey-44g23.5
		ENSGACG00000018746	ENSGACG00000018746
		ENSGACG00000018747	crebbpa
		ENSGACG00000018754	adcy9
27	chrIX 12612422	ENSGACG00000018022	ndst3
		ENSGACG00000018024	ugt8
28	chrIX 13200359	NULL	NULL
29	chrIX 13354585	ENSGACG00000017879	dnaja1
		ENSGACG00000017887	aptx
		ENSGACG00000017889	ENSGACG00000017889
		ENSGACG00000017892	sparcl1
		ENSGACG00000017898	odam
		ENSGACG00000017900	cnga1a
		ENSGACG00000017903	TACR3
30	chrX 4259221	ENSGACG00000002539	atp9b
		ENSGACG00000002474	tekt2
		ENSGACG00000002481	usf2
		ENSGACG00000002484	ENSGACG00000002484
		ENSGACG00000002488	naxe
		ENSGACG00000002503	scn1bb
		ENSGACG00000002516	zbtb22a
		ENSGACG00000002525	ENSGACG00000002525
		ENSGACG00000002526	galr1a
		ENSGACG00000002533	sall3b
31	chrX 10097654	ENSGACG00000005401	PTDSS1
		ENSGACG00000005407	mterf3
		ENSGACG00000005419	uqcrb
		ENSGACG00000005427	lrrc14b
		ENSGACG00000005433	gatad1
		ENSGACG00000005443	ENSGACG00000005443
		ENSGACG00000005445	fbxl2
		ENSGACG00000005492	ENSGACG00000005492
		ENSGACG00000005497	clasp2

		ENSGACG00000005520	ubp1
32	chrX 10555758	ENSGACG00000005895	gabbr2
		ENSGACG00000005879	galnt12
		ENSGACG00000005894	ENSGACG00000005894
33	chrX 15307031	ENSGACG00000008826	ENSGACG00000008826
		ENSGACG00000008831	ENSGACG00000008831
		ENSGACG00000008840	ENSGACG00000008840
		ENSGACG00000008842	ENSGACG00000008842
		ENSGACG00000008844	ENSGACG00000008844
		ENSGACG00000022224	ENSGACG00000022224
		ENSGACG00000021332	ENSGACG00000021332
34	chrXI 6489914	ENSGACG00000008462	tubg1
		ENSGACG00000008473	si:ch211-18i17.2
		ENSGACG00000008483	cntnap1
		ENSGACG00000008492	ezh1
		ENSGACG00000008501	ramp2
		ENSGACG00000008510	ENSGACG00000008510
		ENSGACG00000008514	ENSGACG00000008514
		ENSGACG00000008517	c1ql3b
		ENSGACG00000008519	ccdc43
		ENSGACG00000008523	fzd2
		ENSGACG00000008527	mylk5
		ENSGACG00000008532	si:ch73-141c7.1
		ENSGACG00000008535	hsd17b1
		ENSGACG00000008544	zgc:153952
		ENSGACG00000008553	atp6v0a1a
		ENSGACG00000008605	PTRF
		ENSGACG00000008607	stat3
		ENSGACG00000008634	stat5a
		ENSGACG00000008641	si:ch211-210g13.5
		ENSGACG00000008648	kcnh4a
35	chrXII 5123854	ENSGACG00000012081	prickle3
		ENSGACG00000022195	MIR124-3
		ENSGACG00000012014	ythdf1
		ENSGACG00000012016	ENSGACG00000012016
		ENSGACG00000012020	si:dkey-70p6.1
		ENSGACG00000012022	uckl1a
		ENSGACG00000012046	samd10a
		ENSGACG00000012050	emilin3a
		ENSGACG00000012054	opn7d
		ENSGACG00000012057	snpha
		ENSGACG00000012061	ENSGACG00000012061
		ENSGACG00000012076	fam110a
36	chrXII 6754066	ENSGACG00000011007	plxna2
		ENSGACG00000011016	ENSGACG00000011016
		ENSGACG00000022307	ENSGACG00000022307
		ENSGACG00000021505	ENSGACG00000021505
		ENSGACG00000021506	ENSGACG00000021506
37	chrXII 17793998	ENSGACG00000004001	tns2a
		ENSGACG00000004023	tmem106c
		ENSGACG00000004040	ENSGACG00000004040
		ENSGACG00000004044	sars1
		ENSGACG00000004085	ENSGACG00000004085
		ENSGACG00000004087	fam50a

		ENSGACG00000004113	ENSGACG00000004113
		ENSGACG00000004115	ENSGACG00000004115
		ENSGACG00000004120	ENSGACG00000004120
		ENSGACG00000004125	zgc:103759
		ENSGACG00000004127	gss
38	chrXIII 4342266	ENSGACG00000005923	ENSGACG00000005923
		ENSGACG00000005927	msh3
39	chrXIII 14003722	ENSGACG00000012169	si:dkeyp-14d3.1
		ENSGACG00000012181	slc15a4
		ENSGACG00000012194	glt1d1
40	chrXIII 18701974	ENSGACG00000014606	cnnm4b
		ENSGACG00000014609	ENSGACG00000014609
		ENSGACG00000014611	ENSGACG00000014611
		ENSGACG00000014617	ENSGACG00000014617
		ENSGACG00000014618	h2az2a
		ENSGACG00000014626	hs3st1l2
41	chrXIV 7260047	ENSGACG00000017091	bmp1b
		ENSGACG00000017088	antxr1b
		ENSGACG00000017093	trim69
		ENSGACG00000017101	kcnip3b
		ENSGACG00000017107	ENSGACG00000017107
		ENSGACG00000017108	prnpb
		ENSGACG00000017111	pptc7b
		ENSGACG00000017119	aplnrb
		ENSGACG00000017120	tbc1d10ab
		ENSGACG00000017126	nfkbil1
		ENSGACG00000017132	atp6v0a2a
		ENSGACG00000017144	osbp2
42	chrXV 5954154	ENSGACG00000008383	lrrc9
		ENSGACG00000008300	kif15
		ENSGACG00000008322	tdrd9
		ENSGACG00000008360	ENSGACG00000008360
		ENSGACG00000008396	pcnx4
		ENSGACG00000008408	dhrs7
		ENSGACG00000008424	ppm1aa
43	chrXV 16209873	ENSGACG00000013067	ak7b
		ENSGACG00000013078	ENSGACG00000013078
		ENSGACG00000013081	vrk1
44	chrXVI 4797943	ENSGACG00000002252	ENSGACG00000002252
		ENSGACG00000002254	ENSGACG00000002254
		ENSGACG00000002255	ENSGACG00000002255
		ENSGACG00000002255	ENSGACG00000002255
		ENSGACG00000002259	ENSGACG00000002259
45	chrXVI 6828403	ENSGACG00000002800	il1rapl1a
46	chrXVII 6781353	ENSGACG00000007358	syn2b
		ENSGACG00000007365	timp4.1
		ENSGACG00000007391	ENSGACG00000007391
		ENSGACG00000007398	FOXJ3
		ENSGACG00000007405	ppcs
		ENSGACG00000007417	utp3
		ENSGACG00000007429	ENSGACG00000007429

		ENSGACG00000007430	ENSGACG00000007430
		ENSGACG00000007437	si:dkey-264d12.4
47	chrXX 7959446	ENSGACG00000009832	cd4-1
		ENSGACG00000009781	si:ch211-154o6.3
		ENSGACG00000009790	ENSGACG00000009790
		ENSGACG00000009793	cops7a
		ENSGACG00000009825	si:ch73-86n18.1
		ENSGACG00000009840	usp5
		ENSGACG00000009861	p3h3
		ENSGACG00000009868	gnb3a
		ENSGACG00000009904	ENSGACG00000009904
		ENSGACG00000009907	pex5
		ENSGACG00000009910	clstn3
48	chrXX 8597535	ENSGACG00000009299	abcb4
		ENSGACG00000009330	rundc3b
		ENSGACG00000009361	cnfn
		ENSGACG00000009364	tlr21
		ENSGACG00000009368	pafah1b3
		ENSGACG00000009407	ENSGACG00000009407
		ENSGACG00000009412	ENSGACG00000009412
		ENSGACG00000009423	ENSGACG00000009423
49	chrXX 10599415	ENSGACG00000007546	si:ch73-380l3.2
		ENSGACG00000007557	ENSGACG00000007557
		ENSGACG00000007563	si:dkey-238d18.4
		ENSGACG00000007569	hsc70
		ENSGACG00000007594	ctrl
		ENSGACG00000007597	si:ch211-137j23.7
		ENSGACG00000007600	lin37
		ENSGACG00000007618	ENSGACG00000007618
		ENSGACG00000007622	hspb6
		ENSGACG00000007626	psenen
		ENSGACG00000007639	kmt2bb
		ENSGACG00000007659	igflr1
		ENSGACG00000007664	zbtb32
		ENSGACG00000007668	aplp1
50	chrXXI 16133430	ENSGACG00000000588	cdh12a
		ENSGACG00000000594	CDH10
		ENSGACG00000000591	ENSGACG00000000591
		ENSGACG00000000593	ENSGACG00000000593

FIGURE S1

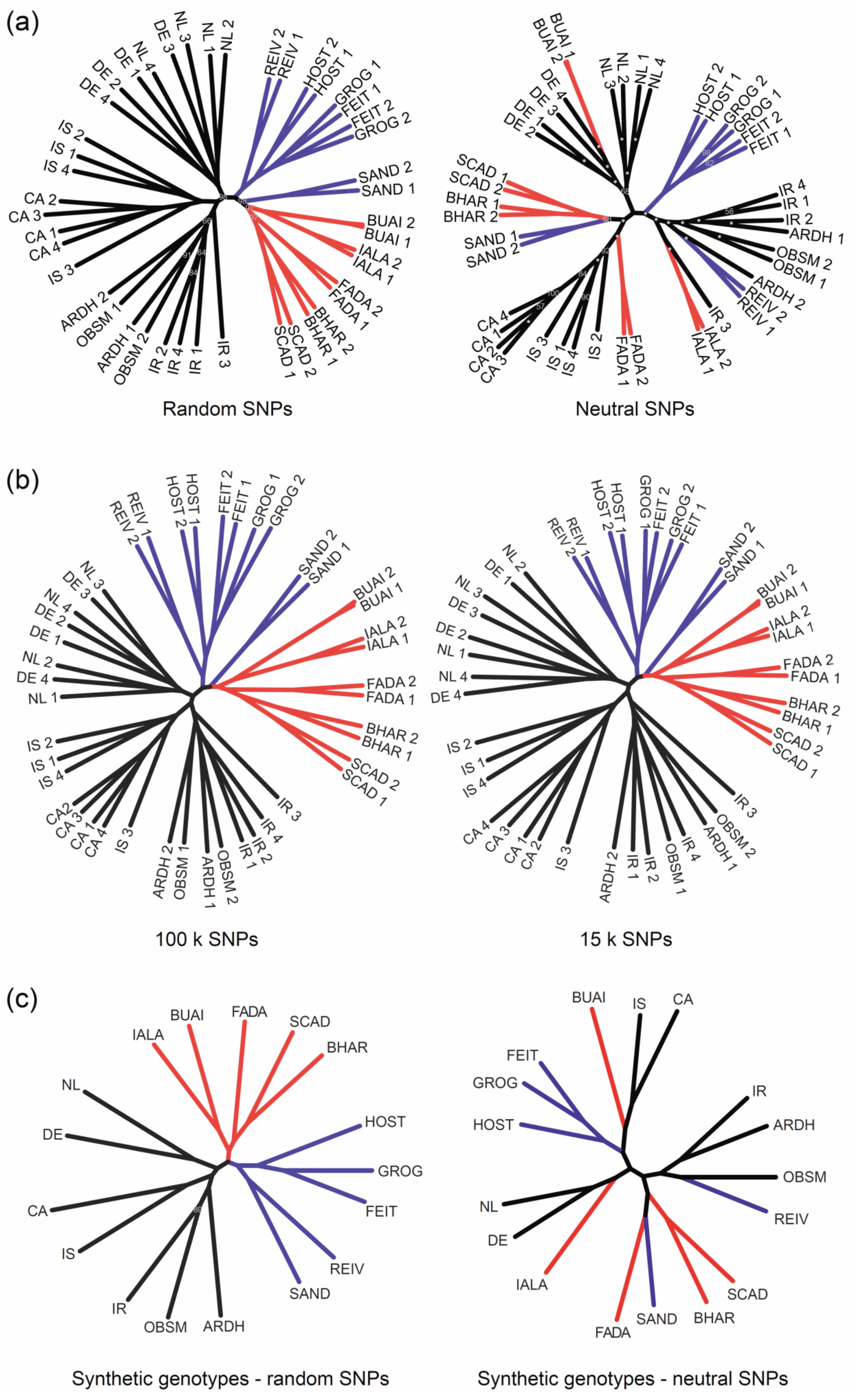

(a)
NL 2
NL 1
NL 3
DE 3
NL 4
DE 1
DE 2
DE 4
IS 2
IS 1
IS 4
CA 2
CA 3
CA 1
CA 4
IS 3
ARDH 2
OBSM 1
ARDH 1
OBSM 2
IR 2
IR 4
IR 1
IR 3
SCAD 1
SCAD 2
BHAR 1
BHAR 2
FADA 1
FADA 2
IALA 1
IALA 2
BUAI 1
BUAI 2
SAND 1
SAND 2
GROG 2
FEIT 2
FEIT 1
GROG 1
HOST 1
HOST 2
REIV 1
REIV 2
Random SNPs
BUAI 1
BUAI 2
NL 3
NL 2
NL 1
NL 4
DE 4
DE 3
DE 1
DE 2
SCAD 1
SCAD 2
BHAR 1
BHAR 2
SAND 1
SAND 2
CA 4
CA 1
CA 2
CA 3
IS 3
IS 1
IS 4
IS 2
FADA 1
FADA 2
IALA 1
IALA 2
IR 3
REIV 1
REIV 2
ARDH 2
OBSM 1
OBSM 2
ARDH 1
IR 2
IR 1
IR 4
HOST 2
HOST 1
GROG 2
GROG 1
FEIT 2
FEIT 1
Neutral SNPs
(b)
REIV 2
REIV 1
HOST 2
HOST 1
FEIT 2
FEIT 1
GROG 1
GROG 2
SAND 2
SAND 1
BUAI 2
BUAI 1
IALA 2
IALA 1
FADA 2
FADA 1
BHAR 2
BHAR 1
SCAD 2
SCAD 1
IR 3
IR 4
IR 2
IR 1
OBSM 2
ARDH 1
OBSM 1
ARDH 2
IS 3
CA 4
CA 1
CA 3
CA2
IS 4
IS 1
IS 2
NL 1
DE 4
NL 2
DE 1
DE 2
NL 4
DE 3
NL 3
100 k SNPs
REIV 2
REIV 1
HOST 2
HOST 1
GROG 1
FEIT 2
GROG 2
FEIT 1
SAND 2
SAND 1
BUAI 2
BUAI 1
IALA 2
IALA 1
FADA 2
FADA 1
BHAR 2
BHAR 1
SCAD 2
SCAD 1
IR 3
OBSM 2
ARDH 1
IR 4
OBSM 1
IR 2
IR 1
ARDH 2
IS 3
CA 2
CA 1
CA 3
CA 4
IS 4
IS 1
IS 2
DE 4
NL 4
NL 1
DE 2
DE 3
NL 3
DE 1
NL 2
15 k SNPs
(c)
IALA
BUAI
FADA
SCAD
BHAR
HOST
GROG
FEIT
REIV
SAND
ARDH
OBSM
IR
IS
CA
DE
NL
Synthetic genotypes - random SNPs
BUAI
IS
CA
FEIT
GROG
HOST
IR
ARDH
OBSM
REIV
SCAD
BHAR
SAND
FADA
IALA
DE
NL
Synthetic genotypes - neutral SNPs

FIGURE S1 (a) Genealogies as presented in Figure 1c, but with bootstrap support based on 500 re-sampling iterations given by gray labels. Unlabeled nodes have 100% support, and nodes marked with a bullet point have support below 50%. (b) Robustness of the observed genealogies, as illustrated by the consistency of the tree topology despite smaller numbers of markers (100,000 and 15,000 SNPs, drawn independently from the full SNP panel). This check is presented for the random SNPs only, but similar topological robustness was observed for the neutral trees. In (c), genealogies are based on synthetic genotypes, as opposed to genotypes from true individuals. The synthetic genotypes were constructed by pooling the individual-level sequence data within each sample site, and drawing a single random nucleotide per site at each SNP. The marker resolution is similar to the one underlying the main genealogies based on individual-level data (random SNPs: n = 200,000, neutral SNPs: n = 120,170).

FIGURE S2

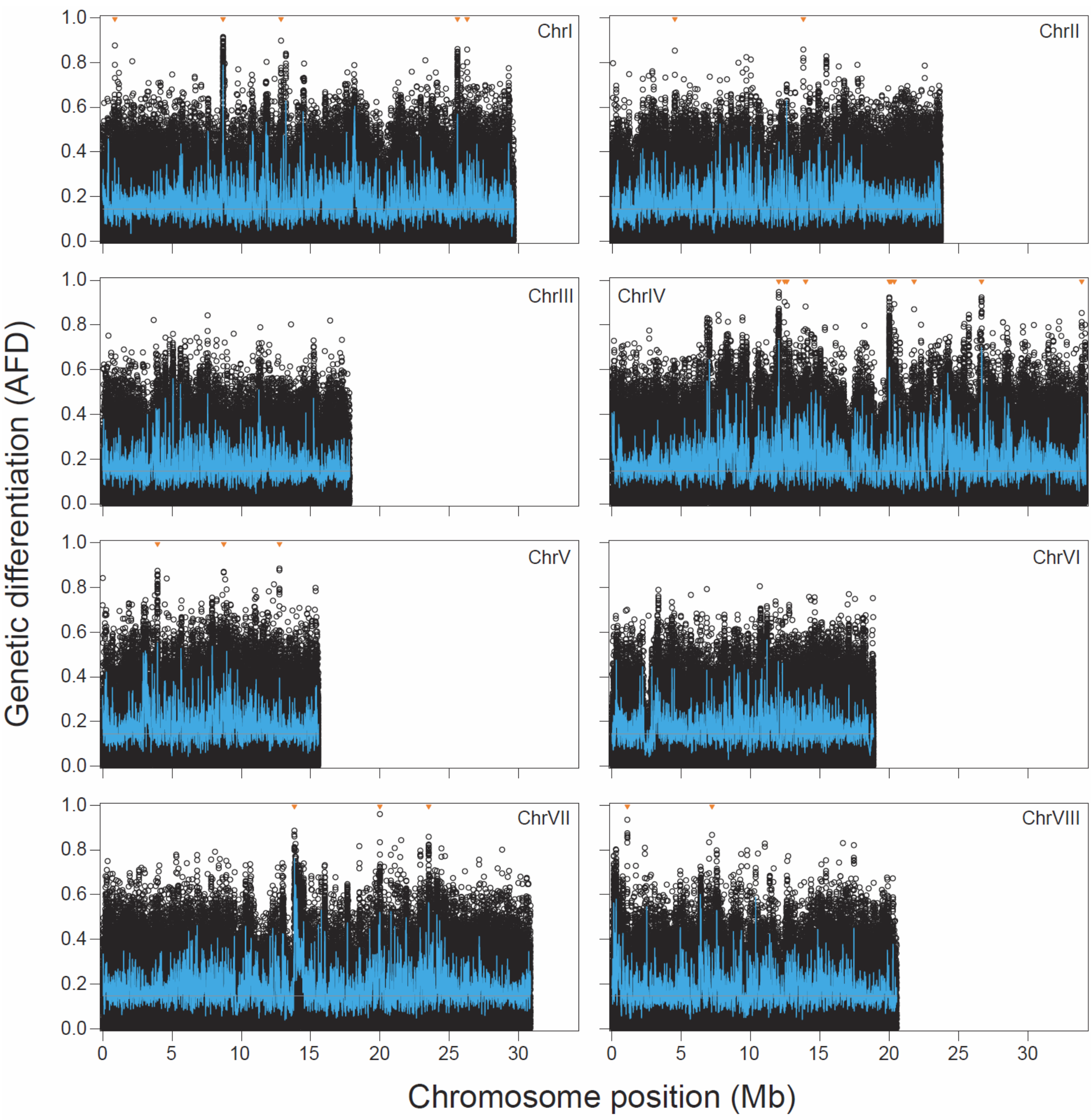

ChrI
ChrII
ChrIII
ChrIV
ChrV
ChrVI
ChrVII
ChrVIII
Genetic differentiation (AFD)
Chromosome position (Mb)
1.0
0.8
0.6
0.4
0.2
0.0
0
5
10
15
20
25
30

FIGURE S2

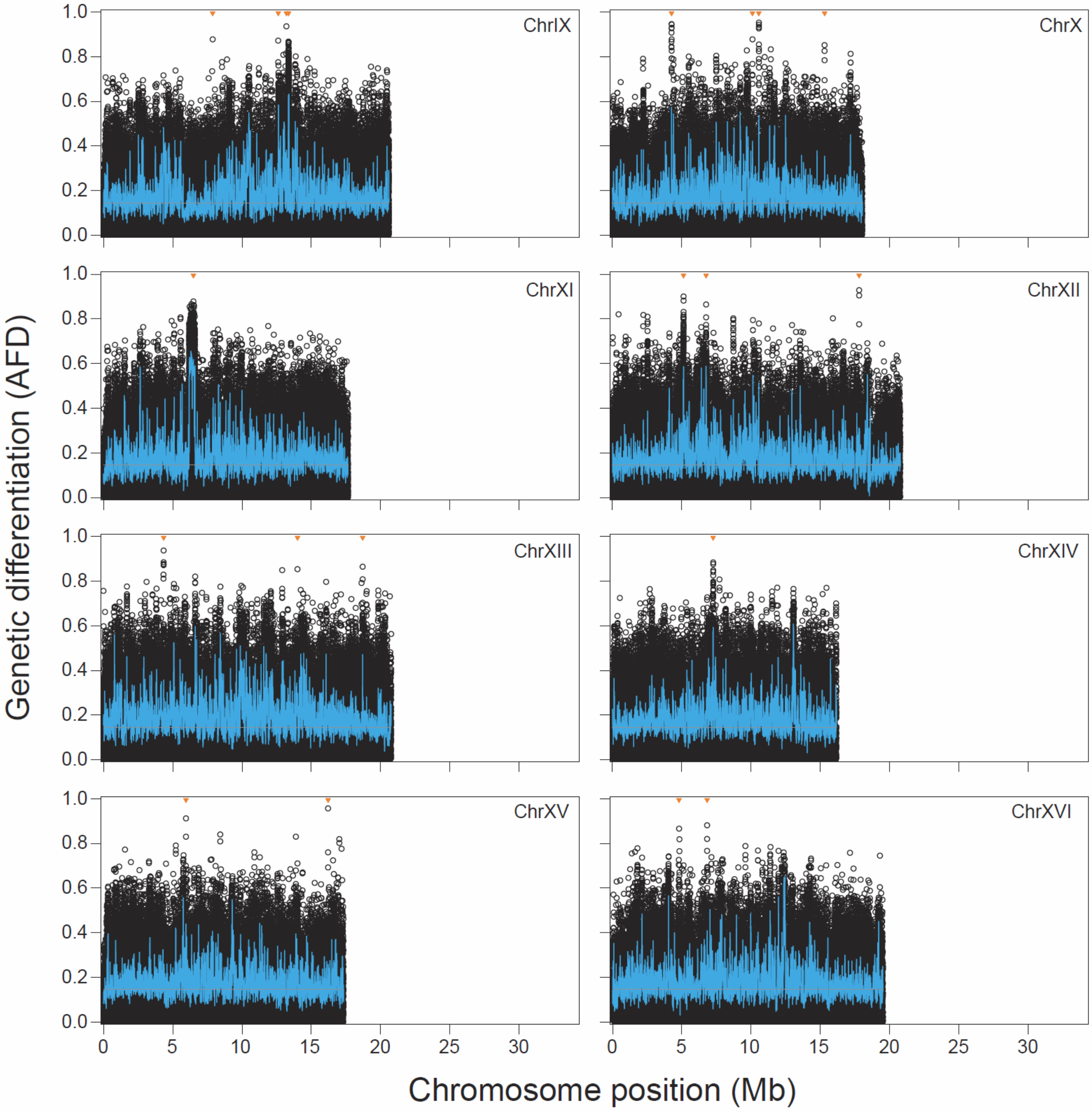

Genetic differentiation (AFD)
Chromosome position (Mb)
ChrIX
ChrX
ChrXI
ChrXII
ChrXIII
ChrXIV
ChrXV
ChrXVI
1.0
0.8
0.6
0.4
0.2
0.0
0
5
10
15
20
25
30

FIGURE S2

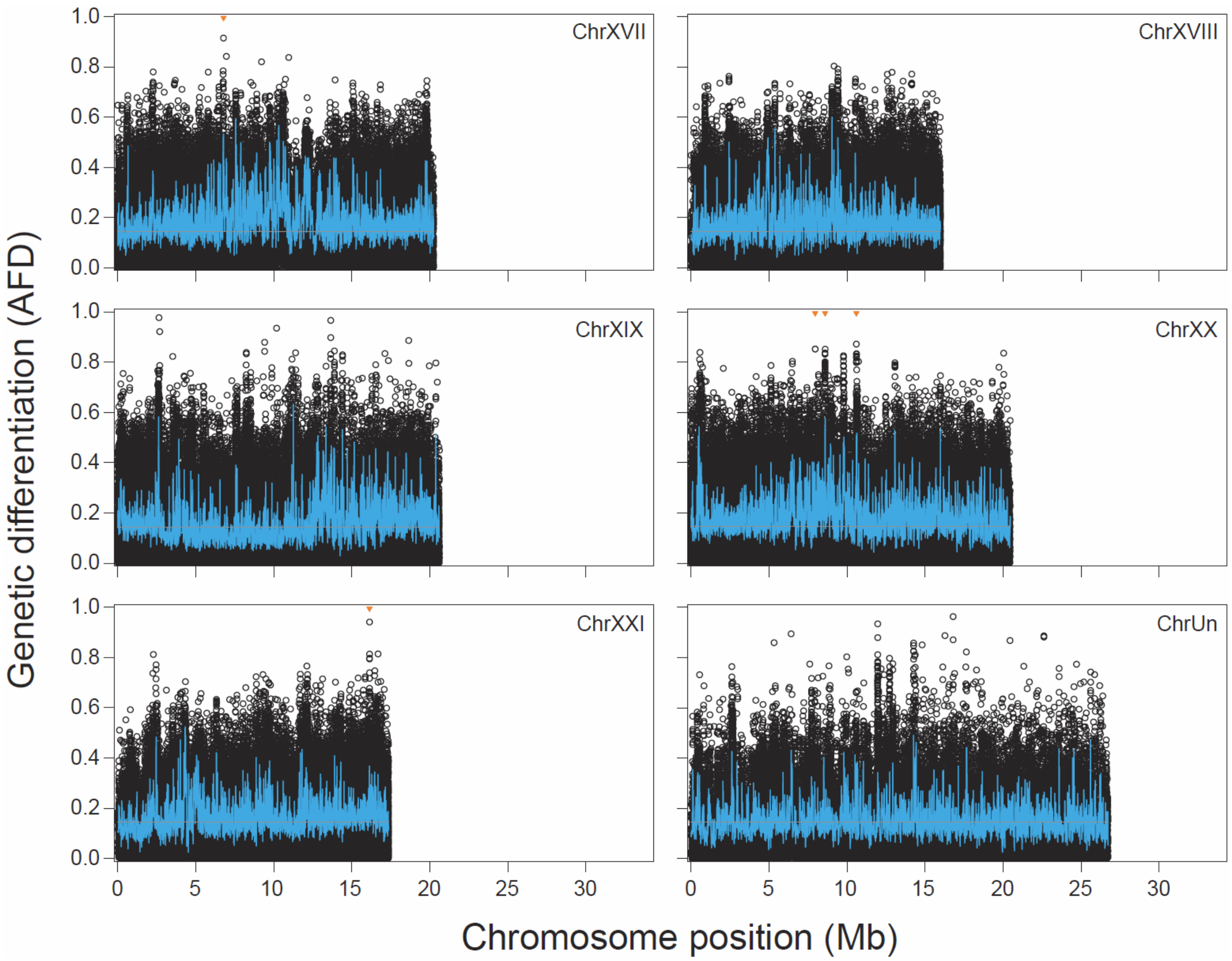

FIGURE S2 Acidic-basic differentiation across the stickleback genome. Shown is the absolute allele frequency difference (AFD) between the acidic and basic sequence pool at individual SNPs (black circles) across all stickleback chromosomes. The blue curves visualize average differentiation across sliding windows of 10 kb with 5 kb overlap (windows with fewer than six SNPs were discarded). The gray horizontal line represents the genome-wide median differentiation (0.145). The orange triangles denote the adaptive SNPs, that is, the markers exhibiting extremely strong and consistent acidic-basic differentiation that were used to explore adaptive standing genetic variation in the marine stickleback samples. Note that the sex chromosome (chrXIX) and the collection of unanchored scaffolds (chrUn) were ignored when selecting the panel of adaptive SNPs.

FIGURE S3

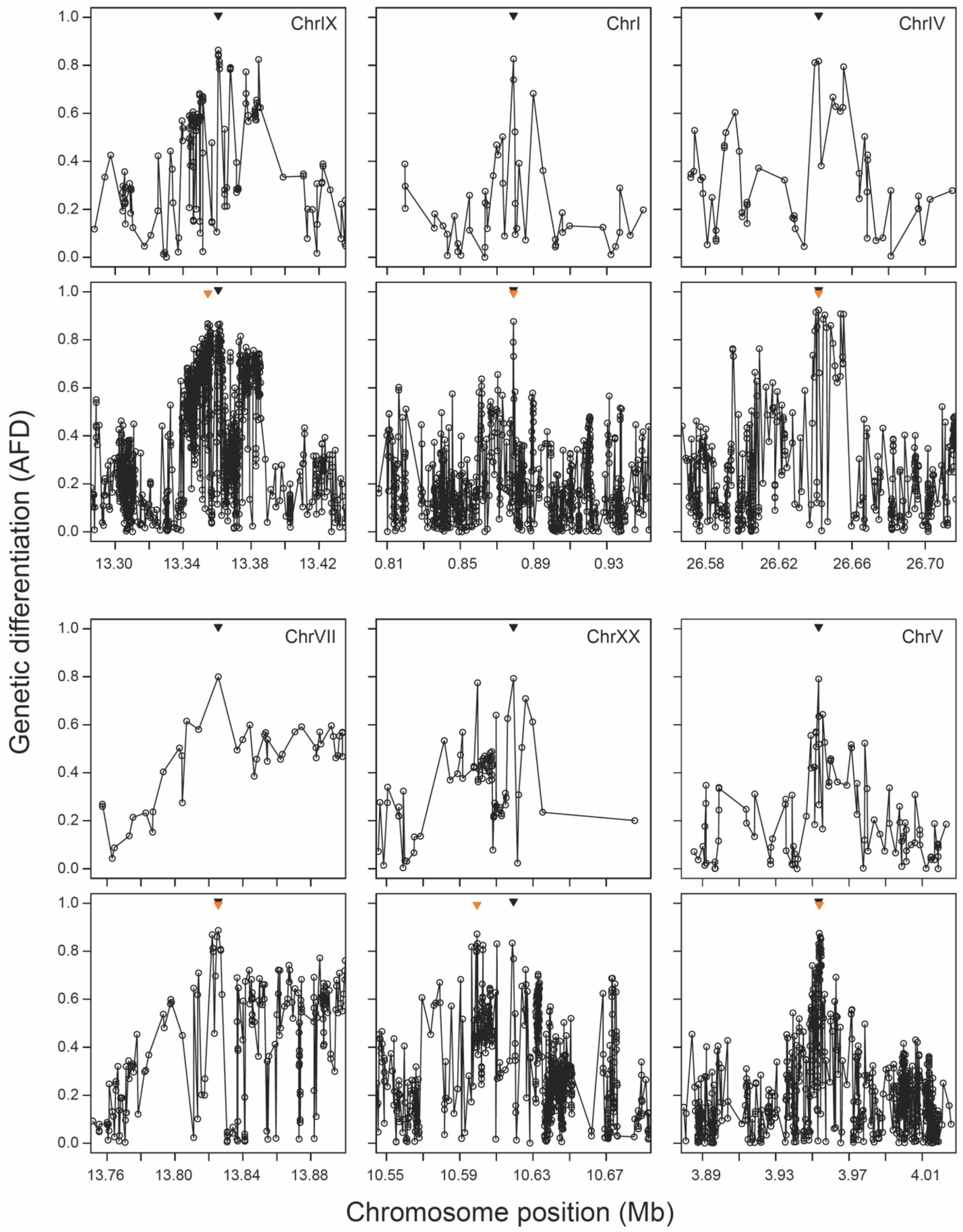
ChrIX
ChrI
ChrIV
ChrVII
ChrXX
ChrV
1.0
0.8
0.6
0.4
0.2
0.0
13.30
13.34
13.38
13.42
0.81
0.85
0.89
0.93
26.58
26.62
26.66
26.70
13.76
13.80
13.84
13.88
10.55
10.59
10.63
10.67
3.89
3.93
3.97
4.01
Genetic differentiation (AFD)
Chromosome position (Mb)

FIGURE S3

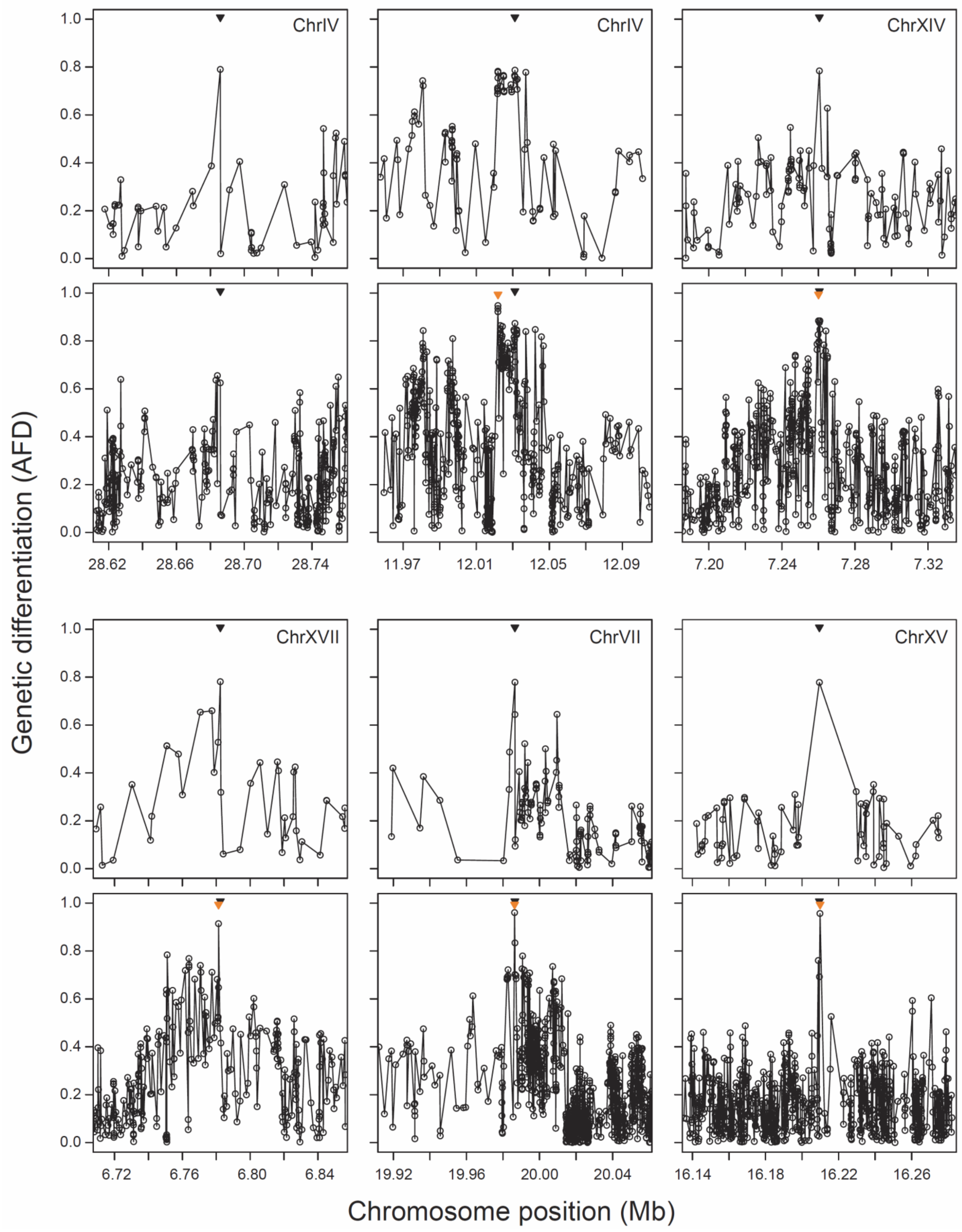
ChrIV
ChrIV
ChrXIV
ChrXVII
ChrVII
ChrXV
1.0
0.8
0.6
0.4
0.2
0.0
28.62
28.66
28.70
28.74
11.97
12.01
12.05
12.09
7.20
7.24
7.28
7.32
6.72
6.76
6.80
6.84
19.92
19.96
20.00
20.04
16.14
16.18
16.22
16.26
Genetic differentiation (AFD)
Chromosome position (Mb)

FIGURE S3

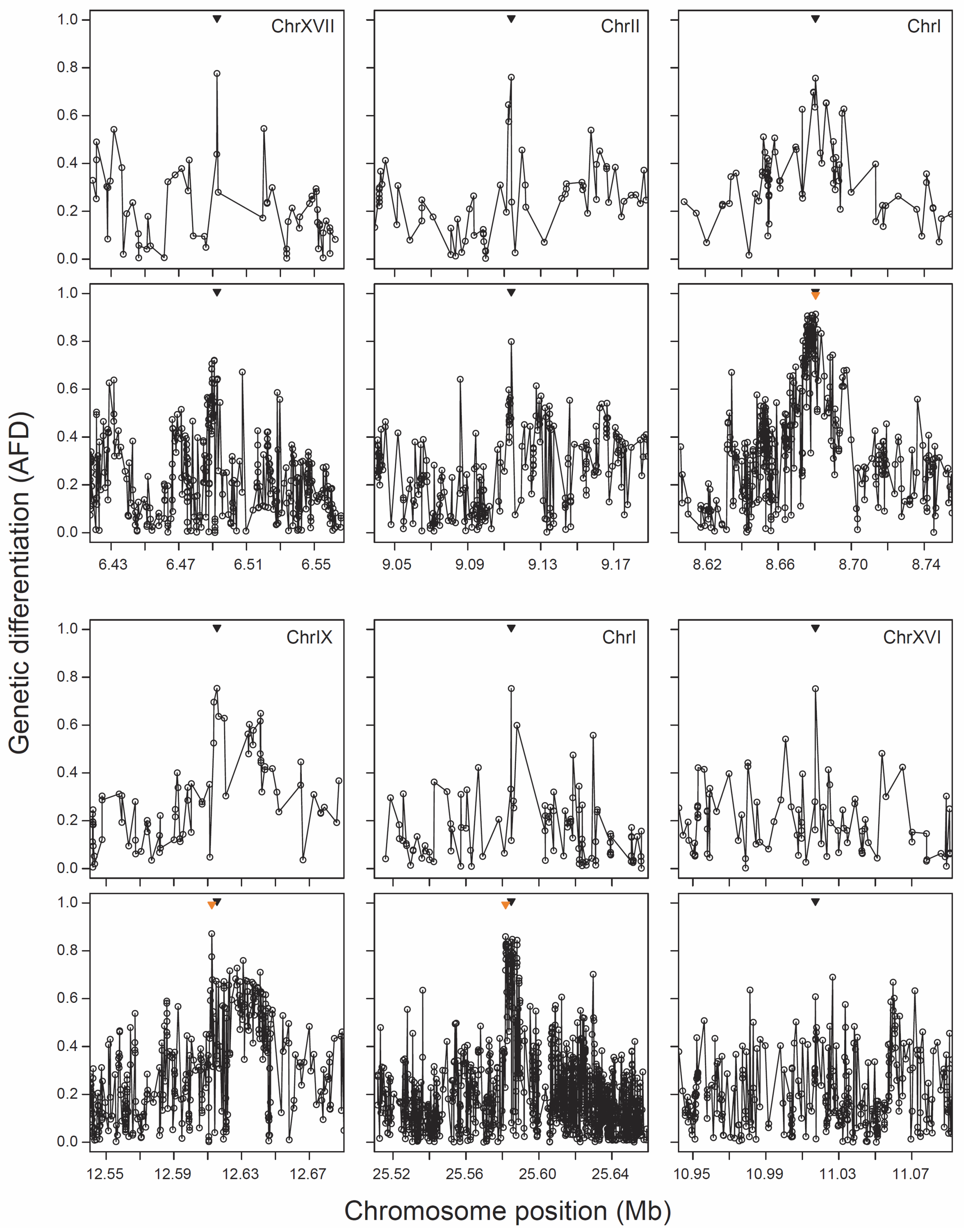

FIGURE S3 Consistency of genome regions identified as important to acidic-basic differentiation between the Haenel et al. 2019 study based on RAD sequencing, and the present work based on whole-genome sequencing. The top panels, labeled with chromosome numbers, present 18 genome regions containing a 'top core SNP' from Haenel et al. 2019, defined as markers displaying an average AFD value of >0.75 across the global acidic-basic comparison in that study.

The panels below show the same chromosome segments, but the data points are from the pooled acidic-basic comparison in the present paper. The top core SNPs from Haenel et al. 2019 are indicated by black triangles in all panels, and when present, the adaptive SNPs from the current study (selection criterion: AFD >=0.85) are added as orange triangles. All chromosome segments are 150 kb wide and centered on the top core SNPs from Haenel et al. 2019, and all tick intervals are 20 kb. Note that Haenel et al. 2019 reports 19 total top core SNPs, but one of them represents the inversion on chromosome XI (also yielding an adaptive SNP in the present study). Because of its large physical size (around 400 kb), this region is not included in this graphic but is visualized comprehensively in Figure 3 of Haenel et al. 2019, and in Figure 4 of the present study. The comparison between the two studies reveals high robustness in the identification of genome regions important to acidic adaptation, despite differences in the analyses used, and partly different panels of underlying individuals. In particular, all top core SNPs from Haenel et al. 2019 represent chromosome segments also showing strong acidic-basic differentiation in the present study. Moreover, most of the top core SNPs from Haenel et al. 2019 reside very close to (median: 424 bp), and sometimes coincide perfectly with, an adaptive SNP from the current study (maximum mismatch observed: 19.9 kb, chromosome XX).

FIGURE S4

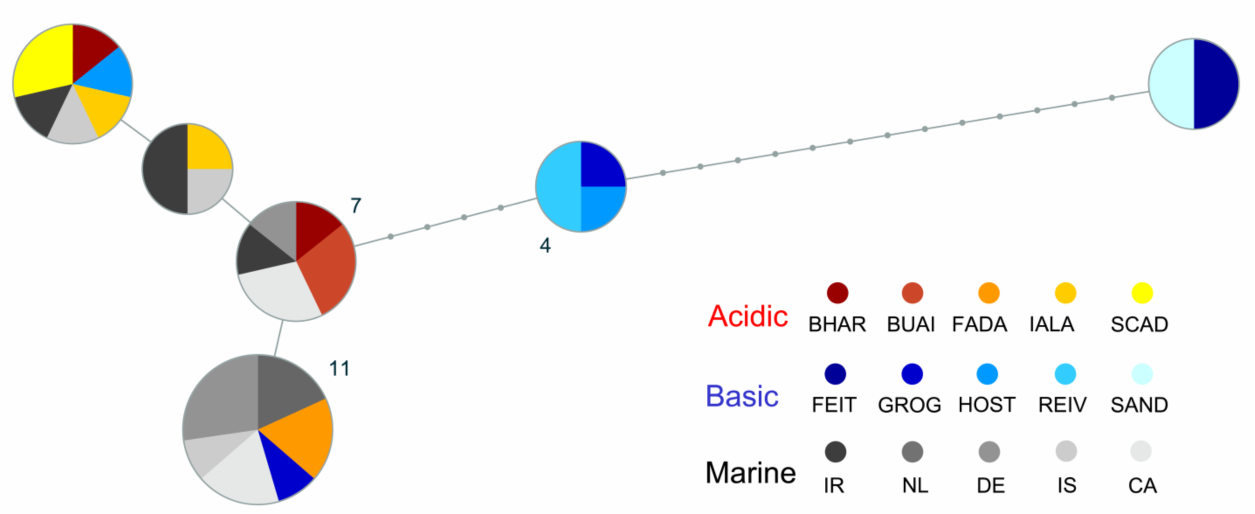

FIGURE S4 Haplotype genealogy based on polymorphisms around an exemplary adaptive SNP (SNP 8 on chromosome IV, Table S3) at which the acidic allele occurs at high frequency (near fixation) in all marine samples. All graphing conventions follow Figure 4b. Note that contrary to most adaptive SNPs, this genome region shows extensive haplotype sharing between acidic and marine stickleback, whereas basic fish harbor distinct haplotypes.

FIGURE S5

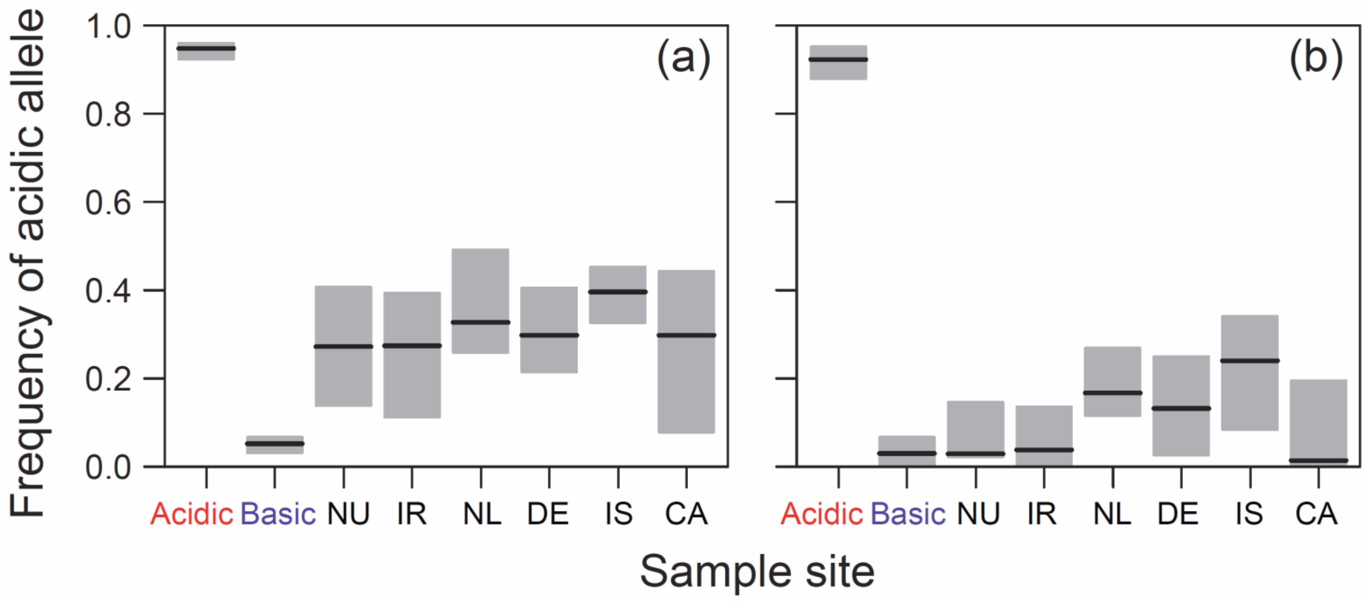

FIGURE S5 Median frequency (black lines) of the acidic alleles in all freshwater and Atlantic marine stickleback sample pools, and the associated 95% compatibility intervals (gray bars) calculated by bootstrap re-sampling allele frequencies within each sample 10,000 times. Analogously to Figure 5, panel (a) is based on all 50 adaptive SNPs, while (b) considers only the 21 adaptive SNPs at which the acidic allele was the minor allele in all marine pools. Note that for both classes of SNPs, the median frequency of the acidic alleles is relatively similar among the marine samples. In particular, the marine sample from North Uist (NU) shows absolutely no indication of elevated allele frequencies compared to the other marine samples.
